# A descriptor guiding the selection of catalyst supports for ammonia synthesis†

**DOI:** 10.1039/d4sc08253b

**Published:** 2025-02-04

**Authors:** Andreas Weilhard, Ilya Popov, Emerson C. Kohlrausch, Gazi N. Aliev, L. Scott Blankenship, Luke T. Norman, Sadegh Ghaderzadeh, Louise Smith, Mark Isaacs, James O'Shea, Anabel E. Lanterna, Wolfgang Theis, David Morgan, Graham J. Hutchings, Elena Besley, Andrei N. Khlobystov, Jesum Alves Fernandes

**Affiliations:** a School of Chemistry, University of Nottingham NG7 2RD Nottingham UK jesum.alvesfernandes@nottingham.ac.uk; b School of Physics & Astronomy, University of Birmingham B15 2TT Birmingham UK; c Cardiff Catalysis Institute, School of Chemistry, Cardiff University CF10 3AT Cardiff UK; d School of Physics and Astronomy, University of Nottingham NG7 2RD Nottingham UK; e Department of Chemistry, University College London London UK

## Abstract

The efforts to increase the active surface area of catalysts led to reduction of metal particle size, down to single metal atoms. This results in increasing importance of support–metal interactions. We demonstrate the mechanisms through which the support influences catalytic activity of nanoclusters: the support electronics, described by the O 2p energy level, and the support surface chemistry, determined by the density of Lewis base sites. Using Ru nanoclusters, our study shows that these parameters can be effectively captured within a single catalyst support descriptor (CSD). The apparent activation energy and turnover frequency (TOF) for the ammonia synthesis correlates strongly with CSD measured for the series Ru/MgO, Ru/Sc_2_O_3_, Ru/CeO_2_, Ru/La_2_O_3_, and Ru/Y_2_O_3_. Furthermore, the study demonstrates that CSD correlates linearly with the binding strength of N–Ru in nanocluster, thereby providing a direct link between the catalyst's surface chemistry and the nature of the support. The catalyst support descriptor developed in this study serves as a simple yet powerful tool for selecting the optimal support material to maximise the activity of metal nanoclusters without altering the metal itself.

## Abstract 2

The efforts to increase the active surface area of catalysts led to reduction of metal particle size, down to single metal atoms.

## Graphical abstract

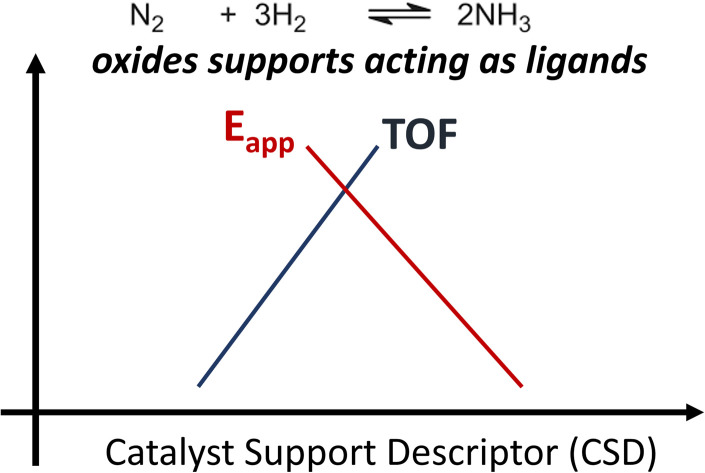

## 1 Introduction

Over the past 20 years, the focus in heterogeneous catalysis has shifted from using bulk metals and particles to nanoclusters and, ultimately, single atoms.^1–3^ This change was driven by the desire to maximise the metal's surface area, which in turn increased the interaction and bonding between the metal and its support due to the sheer increase of the fraction of surface atoms as metal particles become smaller.^4^ As a result, there is now a greater dependence on the electronic structure of catalytic centres on the local environment provided by the support material.^3^ In this context, metal nanoclusters and single atoms can be seen as homogeneous catalysts, where the support functions like a ligand towards the metal centres, strongly modifying their chemical state and thus the activation energy required for a specific reaction.^3,5^

For example, Ru nanoclusters with a size range of 2–3 nm are considered the most effective catalysts for ammonia synthesis because of the maximum concentration of B5 sites.^6–9^ In such cases, improving the catalytic performance depends on changing the electronic environment of Ru catalysts.^7–9^ This can be accomplished by carefully selecting a catalyst support, known as Electronic Metal-Supported Interaction (EMSI).^2^ Numerous reports show support's effect on catalytic performance in various reactions, but there is no clear understanding of the fundamental aspects of this effect,^10–14^ and even more importantly, there are no universal rules to guide the choice of ligand–support for a specific metal. Ideally, such a guide would embed the properties of the support into the classical framework of the Sabatier principle, which relates the binding strength of the crucial intermediate on the catalyst surface to the reaction rate *via* the Bell–Evans–Polanyi principle.^15–19^ Examples include ammonia synthesis (intermediate = surface nitride N*),^7,16,20–25^ CO hydrogenation (intermediate = surface carbonyl CO*)^26^ and CO_2_ hydrogenation (oxygen and carbon binding strengths).^22,27,28^ In ammonia synthesis, the N_2_ activation is generally accepted to be rate-controlling, and consequently, the crucial intermediate is the surface-adsorbed nitride (*N). Assuming a simplified single-atom model, the activation of nitrogen can be mainly driven by the π-backdonation from the filled metal d-orbitals to the π*-orbitals of dinitrogen, which destabilises the N_2_ triple bond. This effect is stronger for the higher-lying d-states of the metal.^23,29,30^ How can higher-lying d-states be achieved without changing the metal, which in turn would decrease the N_2_ activation energy?

In this work, we use the support to achieve this. We developed a catalyst support descriptor (CSD) for ligand–support selection for heterogeneous catalysis based on support band structure and surface chemistry. The CSD was experimentally validated for the ammonia synthesis reaction, the Haber–Bosch process considered the most important chemical reaction in industry. A series of metal oxides CeO_2_, La_2_O_3_, Y_2_O_3_, Sc_2_O_3_, and MgO were investigated due to their differences in the valence band energy, mainly comprised of O 2p, and surface density of Lewis basic sites. Ru nanoclusters of 2–3 nm in diameter were produced by magnetron sputtering, where metal atoms are deposited onto metal oxides' surfaces in vacuum. This enables direct contact between the metal atom catalyst and support surface without any impurities in between, which allows a direct comparison between these materials' activity for an accurate validation of the CSD.

## 2 Results and discussion

**Fig. 1:**
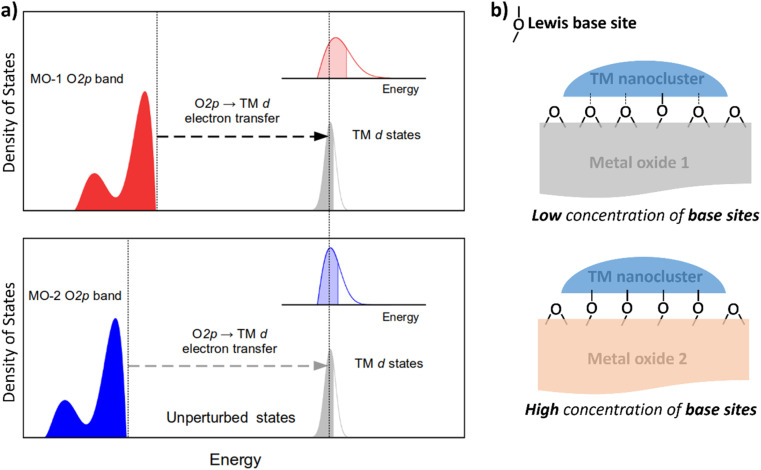
Two main factors affect the electronic structure of metal nanoclusters on metal oxide. (a) Schematic depiction of the density of states of two metal oxides with high (MO-1) and low-lying (MO-2) O 2p bands coupling with the d-band of a transition metal (TM) nanocluster resulting in different degrees of broadening the d-band as shown in the insets. (b) Schematic depiction of Lewis base sites (LBS) interacting with a nanocluster of a transition metal catalyst. Solid lines indicate bonding to LBS, which are defined as binding sites for CO_2_.

Nanocluster–support interactions affecting the electronic structure of d-manifold include two main contributions: (i) pure Coulomb interactions, which do not mix states of the subsystems and are substantial for ionic supports, and (ii) electron transfer, ET (also known as resonance coupling) between band states of the support and d-states of nanoclusters (Fig. 1a). The first contribution provides a constant shift of d-states (crystal field shift), but the effect on the metal internal structure is minor compared to the ET term.^31^ Therefore, ET is the most crucial factor to consider when analyzing the support's effect on catalytic properties.

Because ET can be viewed as a hybridization of orbitals for the transition metal (TM) and the atoms of the support material, its magnitude depends on the energy difference of the states involved and their coupling parameter. In the context of metal oxides, density functional theory and Hartree–Fock calculations have demonstrated that their valence band has a relatively narrow peak just below the Fermi level, with a significant contribution of O 2p atomic orbitals.^32^ Metal oxides' surfaces are terminated by oxygen atoms, whose orbitals are strongly coupled with the d-orbitals of TM nanoclusters. Therefore, the ET from the O 2p band to the TM-d orbital (O 2p band → TM-d) contributes significantly to the resonance interaction between the transition metal nanocluster and the metal oxide support.

To tune the ET contribution for a given transition metal, we must vary the energy of the O 2p band of the supporting oxide. As the energy of the O 2p is lower than the energy of the d-orbitals of the TM, the ET effect will be more prominent for the oxide having the O 2p band located higher on the energy scale (Fig. 1a). The position of the O 2p band in any metal oxide can be experimentally determined from the energy of O-KL_23_L_23_ Auger lines.^33^ The kinetic energy, *E*_kin_, of the O-KL_23_L_23_ line observed in Auger electron spectroscopy (AES) is related to the energy of the O 2p band as:

1 *E*_kin_ = −*E*_1s_ + 2*E*_2p_

where *E*_1s_ < 0 and *E*_2p_ < 0 are the energies of O 1s and O 2p levels in an oxide material measured from the vacuum level. If the energy of low-lying O 1s states, which do not participate in chemical bonding, remains within the range from typical oxides (*ca.* 529–530 eV),^34^ we can estimate the relative position of O 2p bands for any two oxides as2
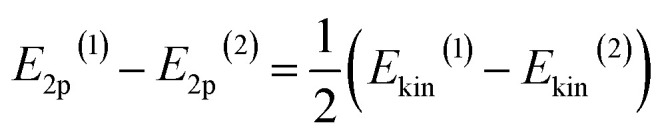


This equation shows that higher kinetic energies of O-KL_23_L_23_ correspond to higher-lying O 2p bands, resulting in a bigger ET effect (Fig. 1a).

In addition to the support's electronic structure, we must consider its surface chemistry. The most relevant aspect is the surface density of Lewis base sites (LBS) responsible for coupling to the nanoclusters' d-states (Fig. 1b). For example, the density of LBS on the support's surface has been shown to be a relatively good descriptor for predicting the catalytic activity of Ru nanoclusters in ammonia synthesis.^35,36^ However, this descriptor works well only for a limited range of oxides and fails for higher concentrations of LBS, such as in Y_2_O_3_.^35,36^

In this work, we unify the two parameters of metal oxides, the energy of the valence band (*E*_2p_) and surface density of LBS (*C*), within a single catalyst support descriptor (CSD), making it applicable to a broader range of metal oxides. Based on the perturbation theory, the Hamiltonian of the metal d-orbitals coupling with O 2p-band is proportional to the concentration of LBS and inversely proportional to their energy difference (see details in ESI, Section 1†). This allows us to propose the following equation for the CSD:3
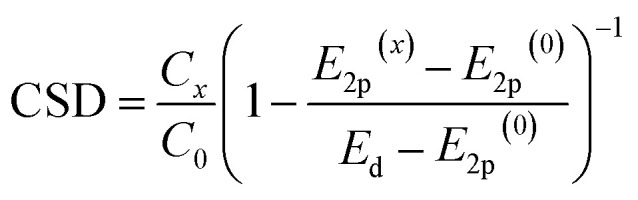
where 0 stands for a reference oxide, *x* for any other oxide in the series, and *E*_d_ is the energy of d-states of the metal nanocluster.

**Fig. 2:**
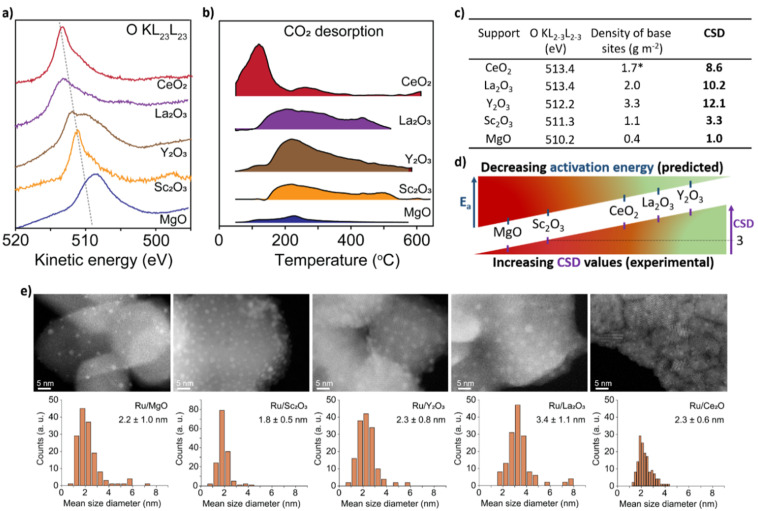
Properties of supports and catalysts. (a) The kinetic energy of the O-KL_23_L_23_ peaks for the metal oxides after signal processing (see details in ESI, Section 2†). (b) CO_2_ desorption normalised by the surface area of the support from the metal oxides measured between 50 °C and 600 °C at atmospheric pressure in a flux of 30 mL per min He (*due to the active redox behaviour of CeO_2_, CO_2_ desorption measurements were carried out after the H_2_ reduction treatment of CeO_2_; see details in ESI, Section 3†). (c) A table containing kinetic energy of O-KL_23_L_23_, the concentration of LBS and the corresponding CSD values calculated according to eqn (3). (d) Schematic depiction of CSD values and predicted *E*_a_. (e) Aberration Corrected Scanning Transmission Electron Microscopy (AC-STEM) of Ru/metal oxide catalysts with size distribution histograms. No sintering of Ru nanoclusters was observed after catalysis (Fig. S18†).

To test CSD, we selected five metal oxides with different stoichiometries (*i.e.* Y_2_O_3_, La_2_O_3_, CeO_2_, Sc_2_O_3_ and MgO), as supports for Ru nanoclusters and investigated their catalytic activity in the ammonia synthesis reaction. To measure *E*_2p_ and concentration of LBS of the supports, we used X-ray photoelectron spectroscopy (XPS) and temperature programmed desorption (TPD) of CO_2_, respectively (Fig. 2a and b, see details in ESI, Sections 2 and 3†). The O-KL_23_L_23_ peak was used to determine the relative values of *E*_2p_ (eqn (2)). XPS measurements show the lowest kinetic energy for MgO (510.2 eV) and the highest for La_2_O_3_ (513.4 eV) (Fig. 2c and Table S1†). MgO was used as a reference point as it has the deepest valence band within this series of supports which allows to estimate *E*_d_–*E*_2p_ which was found 3.15 eV (see details in ESI, Section 1†). Therefore, for MgO CO_2_ TPD measurements, normalised to their surface areas (Table S2†), show that Y_2_O_3_ displays the highest surface density of LBS in the series, which is in line with previous reports,^37^ followed by La_2_O_3_ and CeO_2_, Sc_2_O_3_ and then MgO (Fig. 2c and S12†). Using eqn (3), we calculate the values of the CSD for the catalysts in the series, predicting the following order of the activation energies Ru/Y_2_O_3_ < Ru/La_2_O_3_ < Ru/CeO_2_ < Ru/Sc_2_O_3_ < Ru/MgO (Fig. 2c and d).

To test this prediction and ensure that the observed catalytic parameters are dominated by the effects of supports, it is critical to ensure that the size distributions of Ru nanoclusters are uniform across the metal oxides series. Our methodology, based on the on-surface formation of metal nanoclusters directly from a flux of atoms generated by magnetron sputtering, enables the growth of nanoclusters with accurate size control on different supports.^32–35^ Furthermore, this approach provides direct contact between Ru nanoclusters and the support, without any solvent and impurities associated with traditional wet impregnation methods.^34,36^ Aberration-corrected scanning transmission electron microscopy (AC-STEM) imaging of Ru nanoclusters on the metal oxides after catalysis shows the mean diameter *ca.* 2–3 nm with a narrow size distribution (Fig. 2e), except in Ru@La_2_O_3_ where the average diameter is 3.4 nm. This size range of nanoclusters is optimal for ammonia synthesis because the density of B5 sites is maximised, which are largely responsible for N_2_ activation.^6–9^

**Fig. 3:**
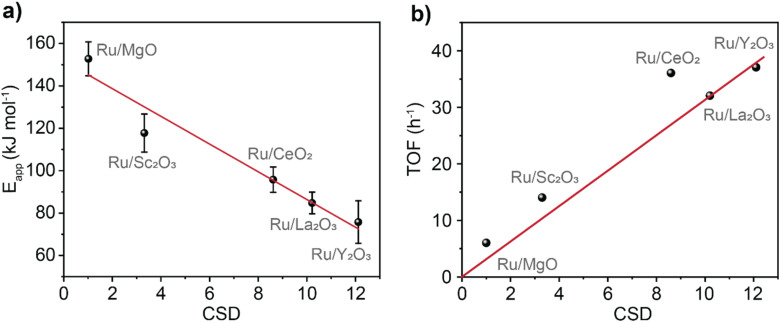
Validation of catalyst support descriptor – CSD. Activation energies (a) and turnover frequency (TOF) at 400 °C (b) for the catalysts plotted against the values of CSD. Catalysts stability was tested over 58 hours of reaction (Fig. S13†). TOF was calculated by dividing the rate of NH_3_ (mmol h^−1^) production by the amount of Ru surface atoms (mmol).

To investigate the electronic effects imposed by the support onto the catalyst, the apparent activation energy, *E*_app,_ of ammonia synthesis was determined (Fig. 3a, see details in ESI†). We found a linear dependence between *E*_app_ and CSD (*R*^2^ = 0.94) with a slope of −20.0 ± 3.5 kJ mol^−1^. This trend captures a wide range of *E*_app_ varying from Ru@MgO (153 kJ mol^−1^ ± 8 kJ mol^−1^) to Ru@Y_2_O_3_ (76 kJ mol^−1^ ± 10 kJ mol^−1^). Noteworthy, the *E*_app_ found for Ru@MgO in our work is in agreement with previous computational predictions for *E*_app_.^38^ Furthermore, an *E*_app_ of *ca.* 70 kJ mol^−1^ (note the similarity to the *E*_app_ found for Ru@Y_2_O_3_ 76 kJ mol^−1^) has been predicted for the Sabatier optimum under low N_2_ conversions.^16,20,39^

Inspecting the turnover frequency (TOF) dependency with CSD, a linear correlation was found for MgO, Sc_2_O_3_, La_2_O_3_ and Y_2_O_3_ (*R*^2^ = 0.99, Fig. 3b). As expected, the lowest turnover frequency is found for Ru/MgO with 6 h^−1^, whilst the highest for Ru/Y_2_O_3_ with 37 h^−1^. Interestingly, Ru/CeO_2_ is an outlier that outperforms its predicted catalytic activity. This effect is most likely due to the reducibility of the CeO_2_ surface (Ce^4+^*vs.* Ce^3+^), mitigating hydrogen poisoning.^40–42^ To elucidate the effect of H_2_ on the catalytic performance, the partial pressure of H_2_ was varied. This does not only give information about H_2_ poisoning but also about the degree of rate control.^20^

**Fig. 4:**
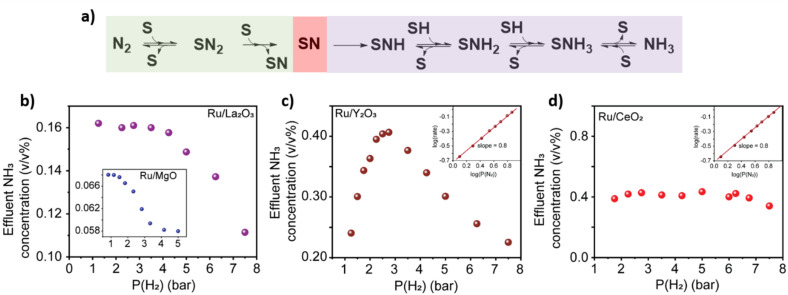
Rate control and H_2_ poisoning mitigation. (a) Schematic depiction of the N_2_ to NH_3_ transformation in the presence of H_2_ on a surface site S. Green highlights the dissociative adsorption of N_2_ onto S, whilst purple highlights the recombinative desorption of NH_3_, and red highlights the surface nitride as the crucial intermediate. (b) Effect of the partial pressure of H_2_ on Ru/La_2_O_3_ and an inset shows the dependence for Ru/MgO. (c) Effect of the partial pressure of H_2_ and N_2_ (inset) onto Ru/Y_2_O_3_. (d) Effect of the partial pressure of H_2_ and N_2_ (inset) onto Ru/CeO_2_. (b–d) All experiments are at 10 bar total pressure a total flow of 40 mL min^−1^ and 400 °C. The total flow was kept constant by the additional flux of Ar in the reaction mixture (see details in ESI, Section 4†).

Upon inspecting the effects of the partial pressure of H_2_ on the reaction rate for Ru/La_2_O_3_, Ru/Sc_2_O_3_ and Ru/MgO we observed a decrease in the rate indicating the dissociative N_2_ adsorption being rate controlling step (Fig. 4a and b and S14†). In contrast, Ru/Y_2_O_3_ exhibits a non-monotonic behaviour indicative of a fluctuation of the rate controlling typical for the Sabatier optimum (see details in ESI, Section 5†). The rate-controlling step fluctuates from dissociative N_2_ adsorption to NH_3_* formation depending on the partial pressure of H_2_ (Fig. 4a and c). Finally, the reaction rate on Ru/CeO_2_ shows no dependence on H_2_ partial pressure (Fig. 4d). This confirms the spillover effect of H* from the nanocluster to the support in Ru/CeO_2_, explaining that this catalyst is an outlier on the TOF *vs.* CSD trend (Fig. 3b).^40,42^ Furthermore, all the metal oxides exhibit N_2_ order close to 1, which confirms the dissociative N_2_ adsorption being rate-controlling under the conditions used in our experiments (Fig. 4c and d insets and S14†).^25^

**Fig. 5:**
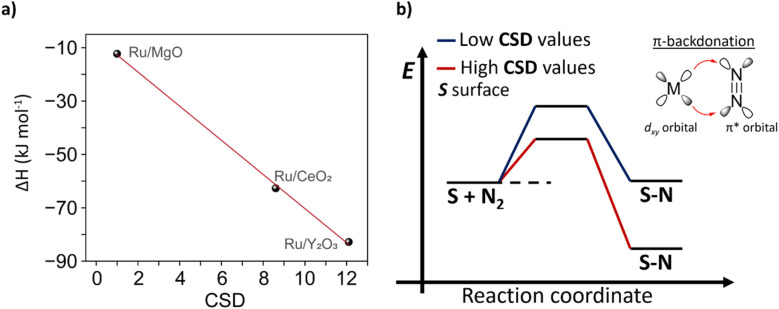
Nitrogen binding strength and catalyst support descriptor – CSD value. (a) The linear relationship between N binding strength and CSD value. (b) The Scheme of the energy profile for N_2_ activation highlights the connection between the CSD value and N binding strength.

To relate CSD with traditional descriptors, such as binding energy of N*, we conducted temperature-programmed adsorption (TPA) and temperature-programmed desorption (TPD) studies (see details in ESI, Section 4†) and estimated thermodynamic parameters for formation N* for Ru/MgO, Ru/CeO_2_ and Ru/Y_2_O_3_ (Tables S4 and S5†). We then demonstrated that CSD correlates linearly with the binding strength and formation enthalpy of N*, *i.e.* higher CSD leads to a more exothermic (and thus exergonic) adsorption of N_2_ (Fig. 5). Consequently, our CSD based on the electronic structure (O 2p band energy) and surface chemistry of the support (LBS surface density) can be linked to previous descriptors, such as the binding strength of N*, and thus regarded as a powerful tool to predict catalytic activity.

## 3 Conclusions

In summary, this work introduces a catalyst support descriptor (CSD) as a powerful tool for optimising support materials in heterogeneous catalysis. By combining the metal oxide O 2p energy level and the surface density of Lewis base sites (LBS), the CSD effectively predicts catalytic performance across a range of metal oxides. We demonstrated a strong correlation between the CSD and catalytic activity, with Ru/Y_2_O_3_ showing the best performance in ammonia synthesis. This work highlights the importance of electron transfer due to resonance coupling in enhancing catalytic activity, offering a unified framework for selecting support materials. By bridging traditional descriptors with the CSD, the study provides a comprehensive methodology for catalyst design, paving the way for advancements in ammonia synthesis and beyond. The CSD offers a novel approach to understanding and improving the electronic environment of catalytic centres, ensuring more efficient and targeted catalyst development This is especially important for the Haber–Bosch process, which is highly energy-intensive and polluting, with high CO_2_ emissions. Our descriptor offers a robust methodology to guide the search for the future efficient catalysts to alleviate the environmental impact of the ammonia synthesis in industry.

## 4 Experimental

### 4.1. General information

MgO, CeO_2_, La_2_O_3_, Sc_2_O_3_ and Y_2_O_3_ were purchased from Sigma Aldrich used without further purification. All solvents were of analytical grade, and all water used in this work was Millipore Milli-Q 18 MΩ ultrapure and deionised. HCl (37% v/v) and HNO_3_ (69% v/v) are ARISTAR™ grade and purchased from VWR Chemicals. Research grade hydrogen (99.999%) and nitrogen (99.999%) was supplied by BOC – LINDE GROUP. Aberration corrected scanning transmission electron microscopy (AC-STEM) measurements were performed using a JEOL 2100F scanning transmission electron microscope with a CEOS aberration corrector operated with an accelerating voltage of 200 kV. Ru nanoclusters were prepared by producing a flow of Ru atoms deposited on metal oxides using magnetron sputtering technique (Table 1).^43,44^ Ru content was measured by Inductively Coupled Plasma Optical Emission Spectrometer (ICP-OES) using a PerkinElmer Optima 2000 DV (Table 1). Catalytic experiments were undertaken in Plug flow reactor provided by micromeritics. Effluent NH_3_ concentrations were measured using GC-TCD analysis provided by Agilent. Microkinetic measurements were measured using CATLAB-PCS Microreactor with an integrated mass spectrometer.

**Table 1:** Magnetron sputtering parameters and obtained Ru content as determined by Inductively Coupled Plasma-Optical Emission Spectrometry (ICP-OES)

Catalyst	Deposition time (min)	Applied power (W)	Ru content (wt%)
Ru/CeO_2_	50	60	1.5
Ru/Y_2_O_3_	40	50	0.8
Ru/La_2_O_3_	40	50	2.0
Ru/MgO	50	60	1.7
Ru/Sc_2_O_3_	40	50	1.7

### 4.2. Metal oxides X-ray photoelectron spectroscopy (XPS) measurements

XPS measurements were performed using Thermo Fisher K-Alpha X-ray Photoelectron Spectrometer using a monochromatic Al K_α_ radiation source operating at 72 W (6 mA × 12 kV) which defines an analysis area of approximately 400 × 400 microns. An analyser pass energy of 160 eV was used for wide energy range survey scans, and 50 eV for elemental regions (C 1s, O 1s, O KLL, Y 3d, La 3d, Ce 3d, Sc 2p and Mg 1s), all samples were recorded using a dual ion-electron charge compensation detector, operating with an argon background pressure of *ca.* 10^−7^ mbar. Samples were mounted by pressing onto silicone-free double-sided adhesive tape. Depth profiling was performed with an Argon ion gun operating at 1000 eV and rastered over a 2 × 1 mm area for 10 seconds per cycle. The data were processed with CASAXPS (Version 2.3.17). The data was charge corrected to the reference C 1s signal at 285.0 eV.

### 4.3. Thermal programmed desorption (TPD) of CO_2_

To measure the concentration of Lewis basic sites (LBS) on the supports, temperature-programmed desorption (TPD) of CO_2_ was performed. The measurements were normalized by metal oxides BET area.

#### 4.3.1 BET area and average particle size

All sample masses were measured using a microbalance with a resolution of 0.0001 g. For isotherm measurements, samples were prepared by degassing under vacuum using a Micromeritics Smart VacPrep at 300 °C for 16 hours. Nitrogen adsorption–desorption isotherms were measured over a relative pressure range of 0.05 to 0.99 at −196 °C using a Micromeritics Tristar. The BET area (*A*_BET_) was calculated from the linear portion of the BET-transform of the isotherm in the relative pressure range of 0.05 to 0.30, with the limits adjusted to achieve a correlation coefficient of at least 0.9999.

Helium pycnometry experiments were conducted on a Micromeritics Accupyc, following degassing at 300 °C for 1 hour using a Micromeritics Smart VacPrep. A total of 200 helium purges were performed at room temperature before conducting 50 volume measurements. The helium density (*ρ*_He_) was determined as the average of these 50 measurements. The average particle diameter (*d*) was calculated from *A*_BET_ and *ρ*_He_, assuming non-agglomerated, non-porous spherical particles according to the formula:
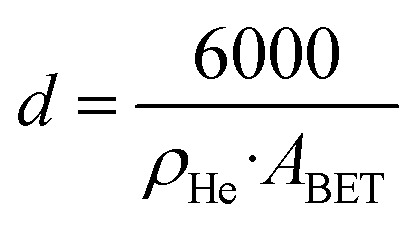


#### 4.3.2 CO_2_ desorption measurements

In a typical experiment the metal oxide is loaded into a quartz tube which in turn is inserted into a CatLab Microreactor provided by Hidden. The sample is held in place by glass wool. After placing the sample in the microreactor, the sample is flushed with He 30 mL min^−1^ and heated to 450 °C with a ramp rated of 10 °C min^−1^ and held to at 450 °C for 6 h. Afterwards, the sample is cooled to 50 °C and the CO_2_ is adsorbed in the absence of He with a flow rate of CO_2_ of 30 mL min^−1^. After 6 h the CO_2_ is replaced by He and after flushing with He for another hour the sample is heated to 950 °C with a ramp rate of 8 °C min^−1^. In the case of CeO_2_, the sample is treated with 5% H_2_ (30 mL min^−1^) in Ar during initial heating to 450 °C and kept under 5% H_2_ in Ar (30 mL min^−1^) for 3 h at 450 °C. Afterwards the sample is flushed with He (30 mL min^−1^) for 3 h and then the sample is cooled to 50 °C. The following adsorption and desorption process are as described above.

### 4.4. Catalytic experiments

Catalytic experiments were conducted in a stainless steel PFR reactor supplied by Micromeritics, PID Microactivity effi, and the effluent NH_3_ concentration was measured using Agilent 8890 GC system. In a typical experiment 1.7 mg of Ru was employed to ensure the same catalyst loading throughout the catalytic experiments. Furthermore, the volume of Ru/metal-oxide support and the employed support were kept constant to keep the contact time the same. The samples were furthermore diluted in 2.3 g of SiC. Both, the catalytic material and the SiC were mixed and loaded into the reactor. The reactor is pressurized to 20 bar and the catalysts are reduced in a flow of H_2_ (30 mL min^−1^) for 6 h at 450 °C. Afterwards, the reactor is cooled to 400 °C and a N_2_ is added to the feed (10 mL min^−1^). After, 10 h samples of the exhaust feed are measured by GC-TCD (Table S3 and Fig. S13†).

To determine the rate orders, all other parameters were kept the same, but the pressure was reduced to 10 bar. To vary the partial pressures of H_2_ and N_2_, Ar was introduced to the feed and the total gas flow was kept constant at 40 mL min^−1^ (Fig. S14†).

The activation energy was measured by changing the total flow of reagents to the reactor at various temperatures. The rate was approximated by measuring the linear regression of the inverse total flow (H_2_ : N_2_ = 3 : 1) against the effluent NH_3_ concentration (Fig. S15†).

### 4.5. N* binding strength

#### 4.5.1 N_2_ desorption of Ru/Y_2_O_3_

In a typical experiment 100 mg Ru/Y_2_O_3_ is loaded into a quartz tube which in turn is inserted into a CatLab Microreactor provided by Hidden. The sample is held in place by glass wool. After placing the sample in the microreactor, the sample is flushed with 5% H_2_ in Ar (30 mL min^−1^) and heated to 450 °C with a ramp rated of 8 °C min^−1^ and held to at 450 °C for 6 h. N_2_ is adsorbed in the absence of any other gas with a flow rate of N_2_ of 30 mL min^−1^. Afterwards the sample is cooled to 50 °C with a ramp rate of 1 °C min^−1^. After 15 h the N_2_ is replaced by He (30 mL min^−1^) and after flushing with He for 5 h the sample is heated to 950 °C with a ramp rate of 8 °C min^−1^.

#### 4.5.2 N_2_ desorption post reaction

In typical experiments 50 mg of the spent catalyst is loaded into a quartz tube which in turn is inserted into a CatLab Microreactor provided by Hidden. The sample is held in place by glass wool. After placing the sample in the microreactor, the sample is flushed with He (30 mL min^−1^) and heated to 950 °C with a ramp rated of 8 °C min^−1^.

#### 4.5.3 N_2_ adsorption measurements

In a typical experiment 150 mg of the investigated sample is loaded into a quartz tube which in turn is inserted into a CatLab Microreactor provided by Hidden. The sample is held in place by glass wool. After placing the sample in the microreactor, the sample is flushed with 5% H_2_ in Ar (30 mL min^−1^) and heated to 450 °C with a ramp rated of 8 °C min^−1^ and held to at 450 °C for 6 h. Then the 5% H_2_ in Ar (30 mL min^−1^) is replaced by He (30 mL min^−1^) and flushed for 6 h. After cooling to 40 °C 5% N_2_ in Ar (5 mL min^−1^) is added to mix. After 15 min the sample is heated to 450 °C with a ramp rate of 1 °C. The adsorption constant *k*_ads_ is calculated assuming that N_2_ adsorbs onto vacant two vacant surface sites.

## Data availability

The data supporting this article have been included as part of the ESI.†

## Author contributions

A. W. performed the catalytic experiments. I. P., S. G. and E. B. were responsible for the theoretical framework. E. K. and L. N. prepared the metal catalysts and performed part of their characterisation. G. N. A. and W. T. performed and analysed the AC-STEM images. L. S. B. and L. S. have performed the base sites and surface area measurements and analysis. M. I., J. O., A. E. L. and D. M. were responsible for the XPS measurements and data analysis. G. J. H, A. N. K. and J. A. F. provided funding acquisition and project supervision. The work was written and edited by all co-authors.

## Conflicts of interest

The authors declare no competing interests.

## Supplementary Material

SC-OLF-D4SC08253B-s001
