# Supplementary material for: A descriptor guiding the selection of catalyst supports for ammonia synthesis
Source: Chem Sci. 2025 Feb 4;16(11):4851–9. doi: 10.1039/d4sc08253b (PMC11808563; doi:10.1039/d4sc08253b)
Supplement: SC-016-D4SC08253B-s001 [file SC-016-D4SC08253B-s001.pdf]

Supplementary Information (SI) for Chemical Science.
This journal is © The Royal Society of Chemistry 2025

Supporting Information

Andreas Weilhard[a], Ilya Popov[a], Emerson C. Kohlrausch[a], Gazimagomed N. Aliev[b], L. Scott Blankenship[a], Luke T. Norman[a], Sadegh Ghaderzadegh[a], Louise R. Smith[c], Mark Isaacs[e], James O'Shea,[d] Anabel Lanterna[a], Wolfgang Theis[b], David J. Morgan[c], Graham J. Hutchings[c], Elena Besley[a], Andrei N. Khlobystov[a] and Jesum Alves Fernandes[a*]

[a]School of Chemistry, University of Nottingham, NG7 2RD, Nottingham, UK

[b]School of Physics & Astronomy, University of Birmingham, B15 2TT, Birmingham, UK

[c] Max Planck-Cardiff Centre on the Fundamentals of Heterogeneous Catalysis FUNCAT, Translational Research Hub, Cardiff University, Maindy Road, Cardiff CF24 4HQ, UK

[d]School of Physics and Astronomy, University of Nottingham, NG7 2RD, Nottingham, UK

[e]Department of Chemistry, University College London, London, UK

1. Catalyst support descriptor (CSD) theoretical description

Resonance interactions between oxides and d-states of nanocluster

Resonance contribution to the Hamiltonian of the d-states of a nanocluster can be expressed previously:[1]

$$H^{res}_{\mu\nu} = \sum_{i,j} \beta_{\mu i}\beta_{\nu j} \sum_{n,k} \frac{\langle i \mid nk \rangle \langle nk \mid j \rangle}{E_d - E_{nk}} \qquad Equation\ S1$$

where indices *μ* and *ν* correspond to the d-states, *i* and *j* enumerate atomic orbitals of the support, summation over ***n, k*** goes over band states of the support, E_d – is energy of the d-states and $E_{n\mathbf{k}}$ – band energy.

Exact calculation of the resonance contributions requires detailed knowledge of the electronic structure of the support and d-states of the nanocluster. However, qualitative analysis of this equation allows to suggest functional form of a descriptor reflecting the magnitude of electron transfer between support and transition metal clusters. To do that we make the following approximations:

1. The most significant contribution to the electron transfer comes from interaction of the d-states with the frontier states of the valence band, which mostly consist of O2p in the case of oxides. It allows to restrict summation over all atomic orbitals of the support to the summation over oxygen 2p orbitals.

2. Density of states of the O2p valence band typically looks like a narrow peak, therefore we use a constant value of energy for all band states $E_{n\mathbf{k}} = E_{2p}$.

3. Typically, non-diagonal orbital-projected density matrix elements are much smaller compared to the diagonal one, which allows to omit all the terms whith i ≠ j.

4. We assume that all oxygen 2p atomic orbitals are equivalent and have the same coupling with the d-states of the nanocluters (mean field approximation).

These approximations allow to simplify the equation for the resonance contribution:

$$H^{res}_{\mu\nu} \approx \sum_{i} \beta_{\mu i}\beta_{\nu i} \frac{P_{ii}}{E_d - E_{2p}} = const \sum_{i} \frac{1}{E_d - E_{2p}} \qquad Equation\ S2$$

where the summation now goes over all 2p orbitals of oxygen interacting with the nanocluster. The sum is proportional to the number of these orbitals, hence proportional to the surface density of the base sites. These considerations yield the equation (3) of the manuscript.

2. X-ray photoelectron spectroscopy (XPS) measurements

X-ray photoelectron spectroscopy (XPS) measurements were conducted for CeO_2 using Thermo Scientific K-Alpha instrument, with no pre-treatment applied to the samples prior to the measurements. The data were processed with CASAXPS (Version 2.3.17). The data was charge corrected to the reference C 1s signal at 285.0 eV.

The XPS fittings were performed using the following parameters:

- O 1s - LA(1.53,243) line shape, Shirley background, full width at half maximum (FWHM) was kept below 3 eV;
- O KLL - LA(1.53,243) line shape, linear background, full width at half maximum (FWHM) was kept below 4 eV.
- The O 1s peak signal can be divided in three signals: lattice oxygen atoms with a binding energy generally in the range of 529–530 eV (1); then a second peak between 530-532 eV (2) assigned as hydroxide and then 532-533 eV (3) to carbonate species.[2] Therefore, we constrain the position of the peak (2) to be between 1-2 eV from peak (1); and peak (3) to be 2-3 eV from peak (1). For the O KLL, same approach was used with a constrain of the position of peak (2) to be 2-3 eV from peak (1), and peak (3) to be 3-4 eV from peak (1). In a few instances, a peak at a higher binding energy than 533 eV appears, which some reports attribute to adsorbed water. However, as this remains a topic of ongoing debate, it is not addressed in this work, as it falls outside the scope of our focus.

CeO$_2$ support XPS measurements

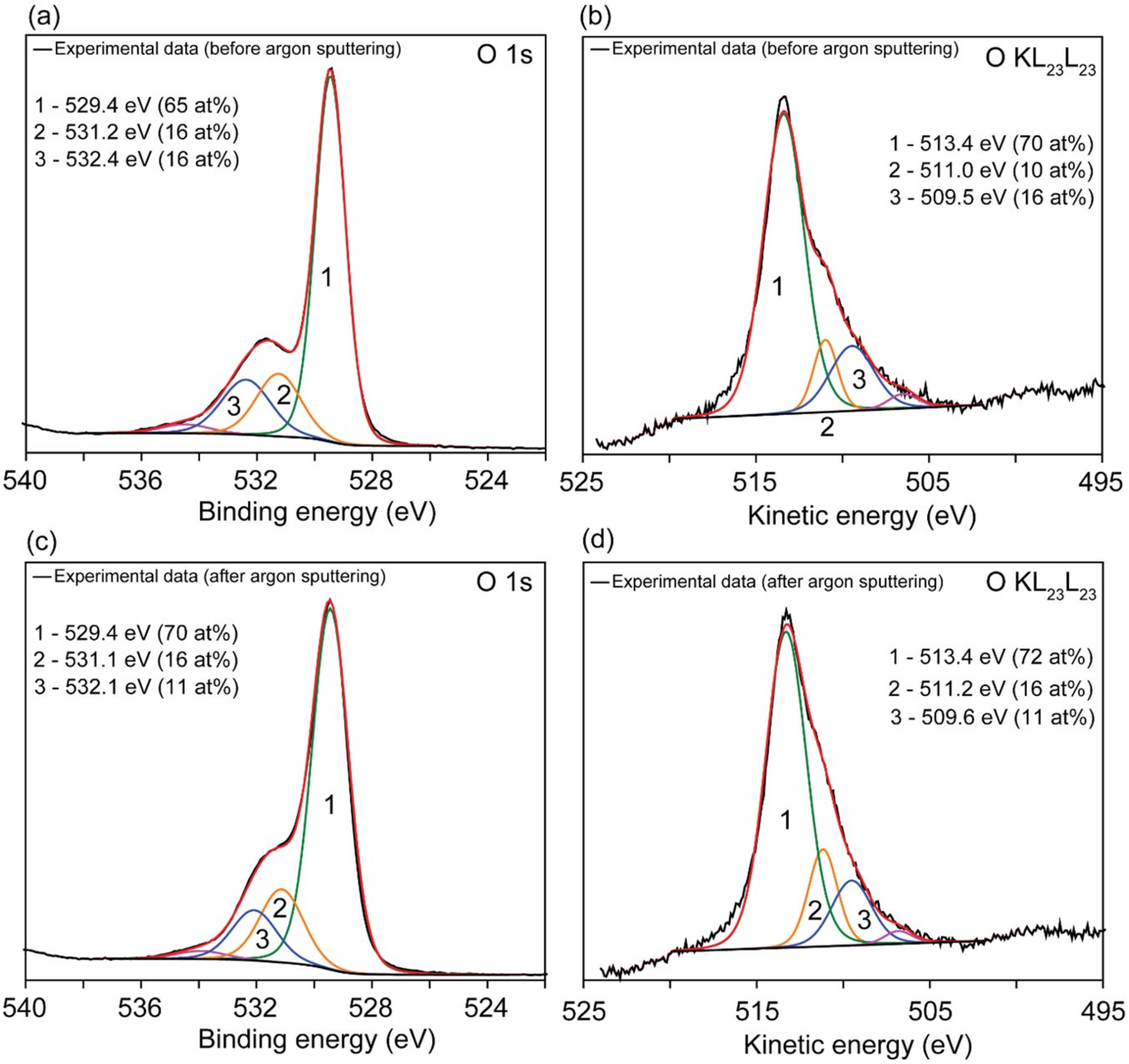

Figure S1. X-ray photoelectron spectroscopy (XPS) measurements for CeO$_2$ were analysed before **a-b)** and after **c-d)** CeO$_2$ surface argon sputtering. O 1s and O KLL spectra after argon sputtering show results very similar to those before argon sputtering, with a slight increase on oxygen lattice signal as expected for CeO$_2$.

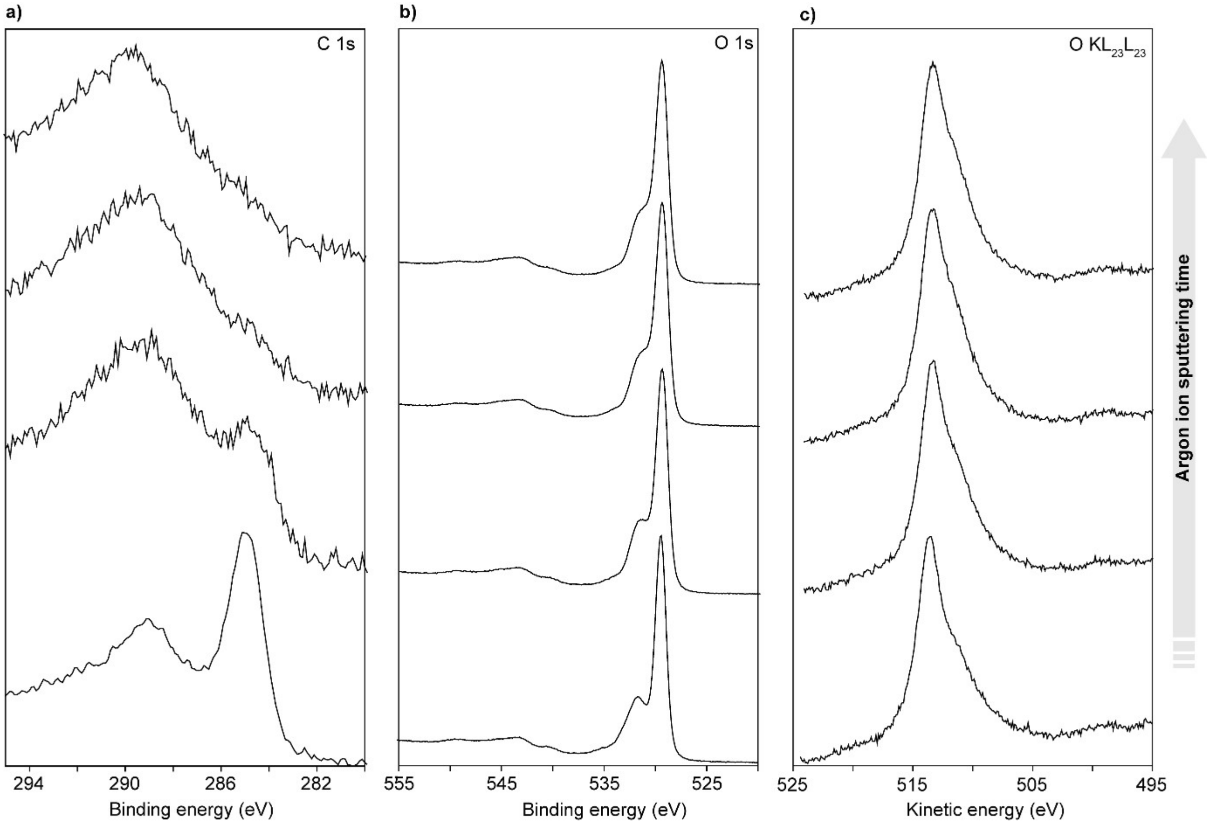

Figure S2. X-ray photoelectron spectroscopy (XPS) measurements for CeO_2 to observe the effect of surface argon sputtering over time for **a-c)** C 1s, O 1s, and O KLL. The aim of these measurements was to confirm the assignment of lattice oxygen, hydroxide and carbonate species, as the surface argon sputtering process should completely or partially remove hydroxide and carbonate species, resulting in a decrease of these two species after argon sputtering and an increase in the lattice oxygen signal. The bottom measurement of each figure shows the spectra before argon sputtering, used as a reference. It is important to highlight that in the case of CeO_2, lattice oxygen is the predominant component even without argon sputtering, so no major changes were expected. After the first argon sputtering cycle, a slight decrease in the signal of the peak at the higher binding energy component is observed for the O 1s spectra. This indicates that the signal of the peak associated with hydroxide and carbonate species is slightly lower after the initial argon sputtering, which is also observed in the O KLL spectra.

La$_2$O$_3$ support XPS measurements

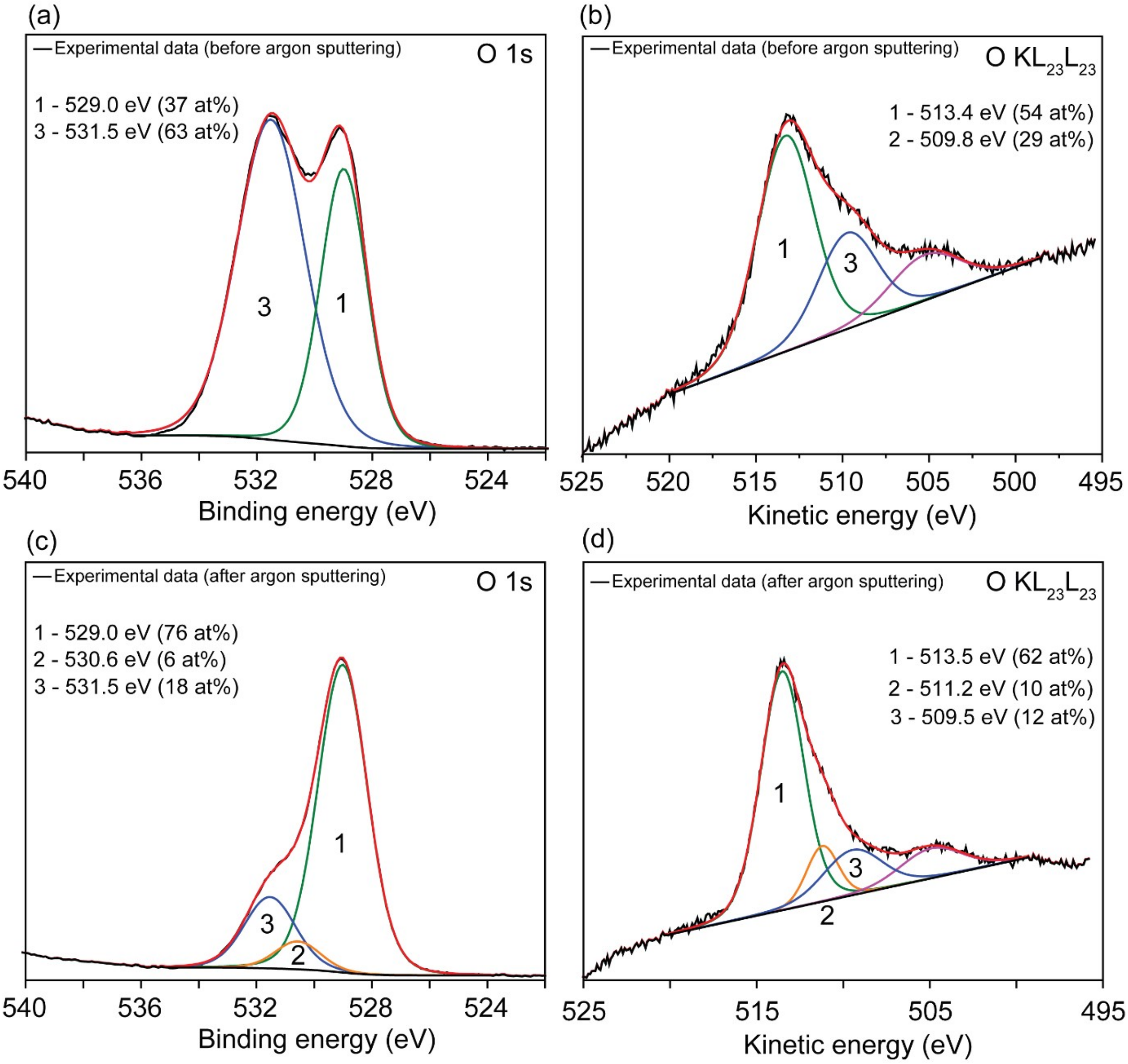

Figure S3. X-ray photoelectron spectroscopy (XPS) measurements for La$_2$O$_3$ were analysed before **a-b)** and after **c-d)** La$_2$O$_3$ surface argon sputtering. Before argon sputtering, the O 1s and O KLL spectra indicated that carbonate was the major component. However, after sputtering, the spectra revealed that lattice oxygen became the primary component, with a minor formation of hydroxide species on the La$_2$O$_3$ surface. The peak at low kinetic energy, located near the carbonate species, leads to an overestimation of the lattice oxygen concentration in the O KLL spectra. However, it does not affect the peak position of lattice oxygen, as demonstrated after argon sputtering, where lattice oxygen became the major component. The low kinetic energy peak, which is characteristic of La$_2$O$_3$[3], lies outside the scope of this study, and its nature is therefore not discussed in detail.

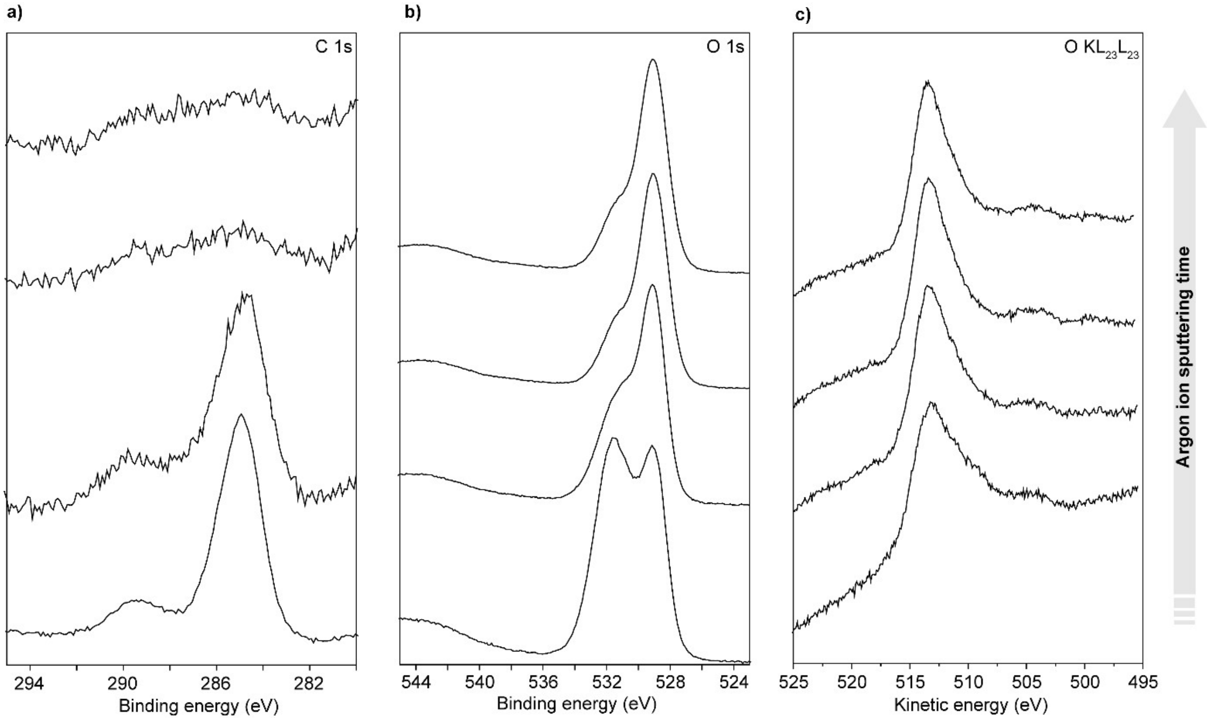

Figure S5. X-ray photoelectron spectroscopy (XPS) measurements for La_2O_3 to observe the effect of surface argon sputtering over time for **a-c)** C 1s, O 1s, and O KLL. The aim of these measurements was to confirm the assignment of lattice oxygen, hydroxide and carbonate species, as the surface argon sputtering process should completely or partially remove hydroxide and carbonate species, resulting in a decrease of these two species after argon sputtering and an increase in the lattice oxygen signal. The bottom measurement of each figure shows the spectra before argon sputtering, used as a reference. A significant change in the height of the major peaks between the lower and higher binding energy components is observed for the O 1s spectrum after the first argon sputtering cycle. This indicates that lattice oxygen becomes the major component following the initial argon sputtering cycle. This behaviour is also observed in the O KLL spectrum.

Y_2O_3 support XPS measurements

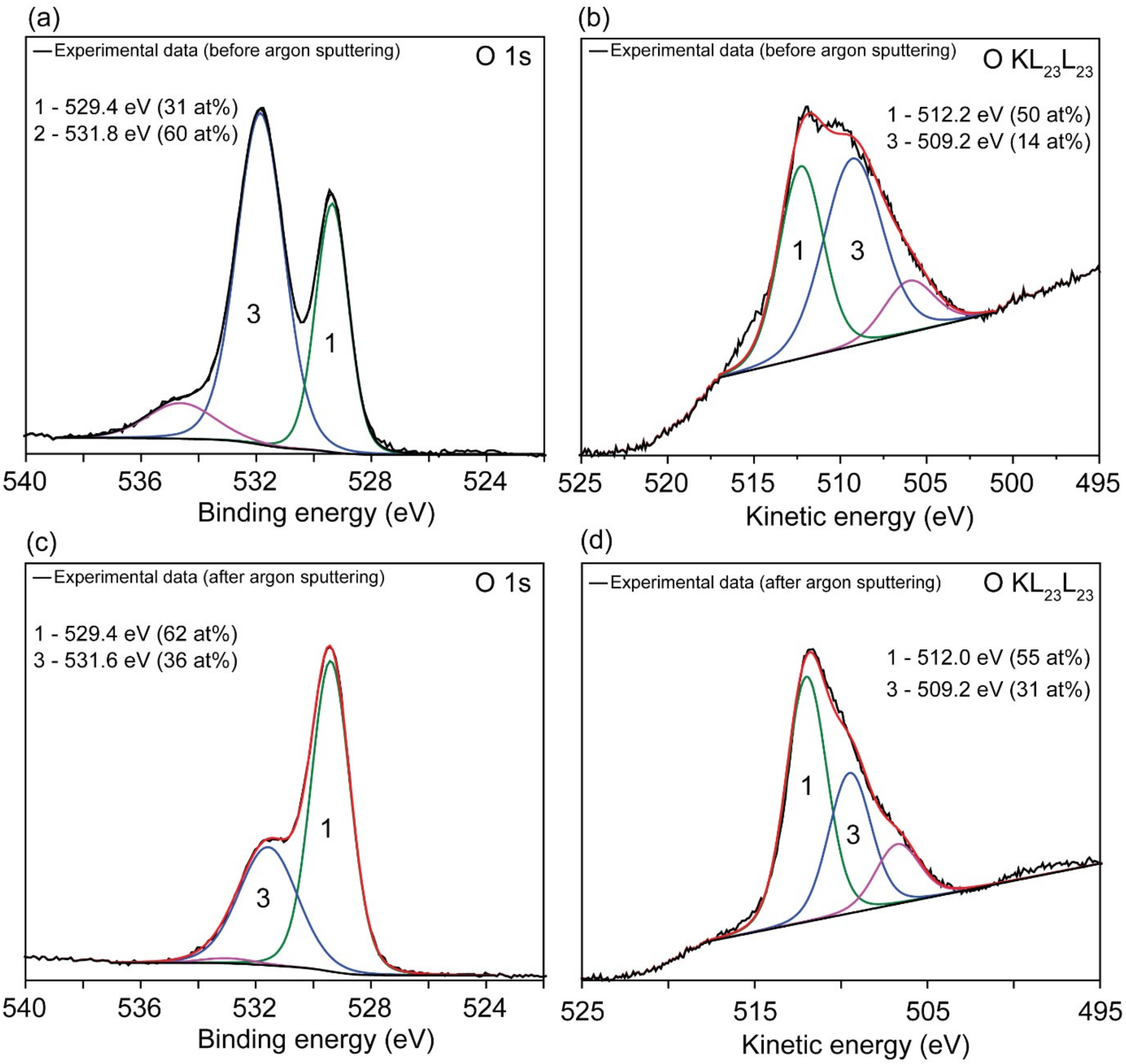

Figure S6. X-ray photoelectron spectroscopy (XPS) measurements for Y_2O_3 were analysed before **a-b)** and after **c-d)** Y_2O_3 surface argon sputtering. Before argon sputtering, the O 1s and O KLL spectra indicated that carbonate was the major component. However, after sputtering, the spectra revealed that lattice oxygen became the primary component.

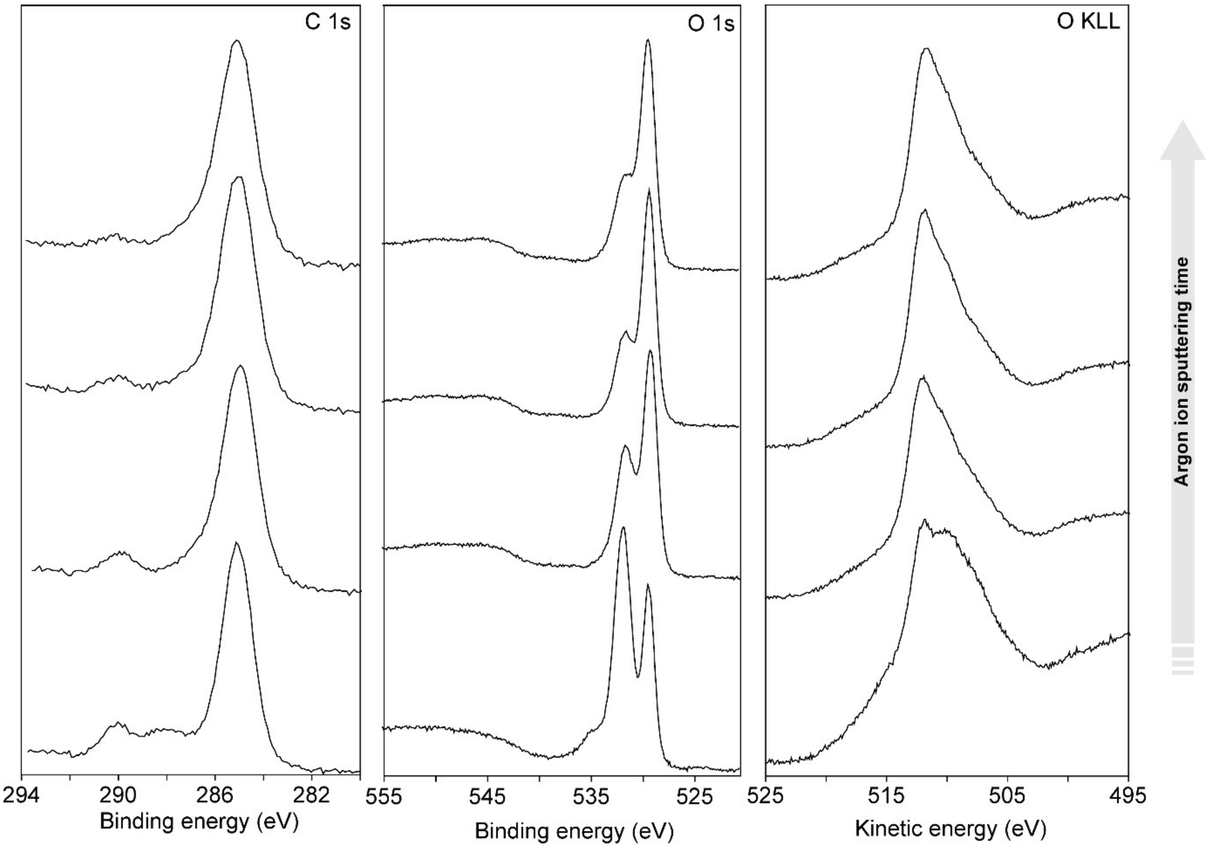

Figure S7. X-ray photoelectron spectroscopy (XPS) measurements for Y_2O_3 to observe the effect of surface argon sputtering over time for **a-c)** C 1s, O 1s, and O KLL. The aim of these measurements was to confirm the assignment of lattice oxygen, hydroxide and carbonate species, as the surface argon sputtering process should completely or partially remove hydroxide and carbonate species, resulting in a decrease of these two species after argon sputtering and an increase in the lattice oxygen signal. The bottom measurement of each figure shows the spectra before argon sputtering, used as a reference. A significant change in the height of the major peaks between the lower and higher binding energy components is observed for the O 1s spectrum after the first argon sputtering cycle. This indicates that lattice oxygen becomes the major component following the initial argon sputtering cycle. This behaviour is also observed in the O KLL spectrum.

MgO support XPS measurements

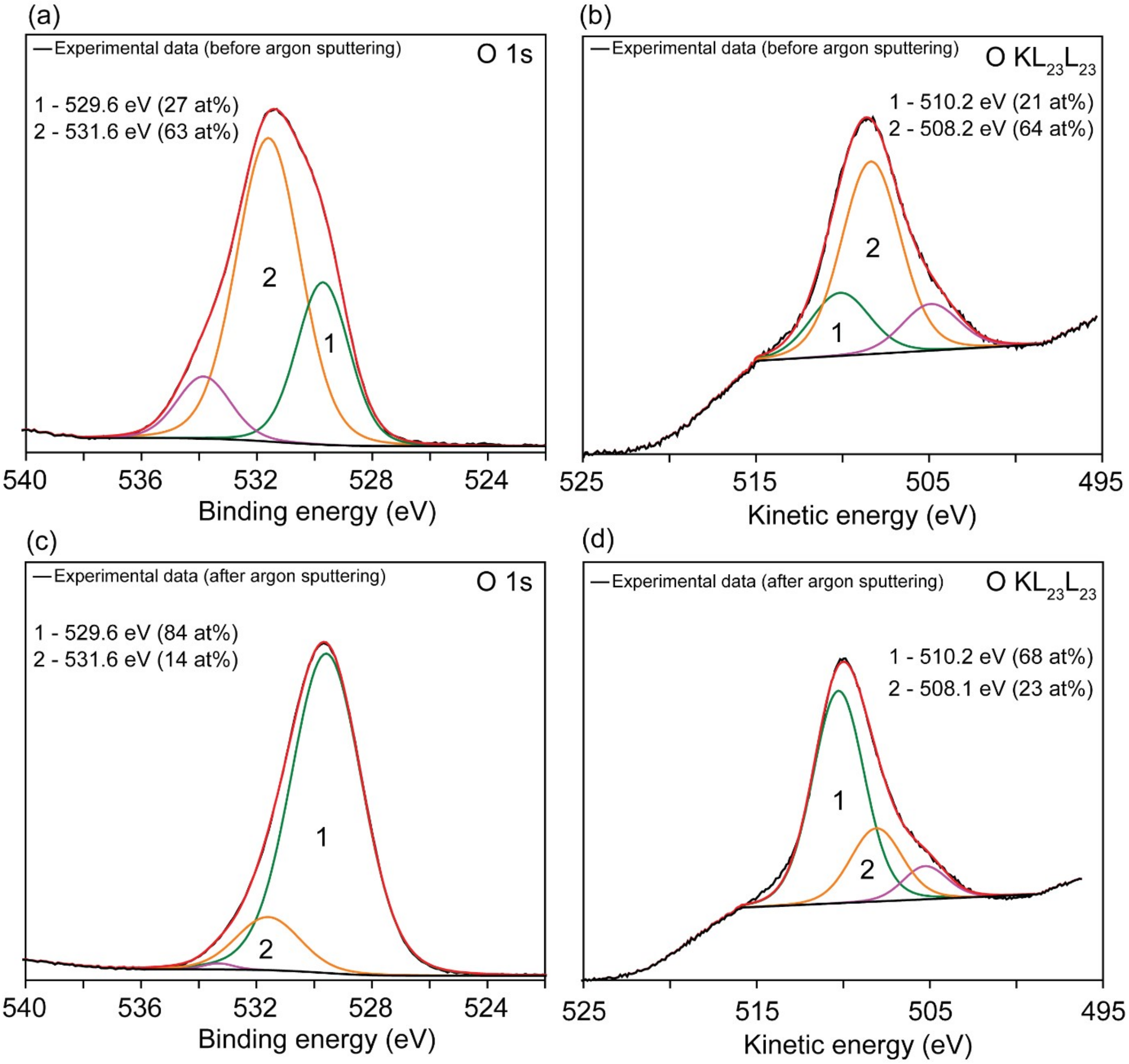

Figure S8. X-ray photoelectron spectroscopy (XPS) measurements for MgO were analysed before **a-b)** and after **c-d)** MgO surface argon sputtering. Before argon sputtering, the O 1s and O KLL spectra indicated that hydroxide species were the major component. However, after sputtering, the spectra revealed that lattice oxygen became the primary component.

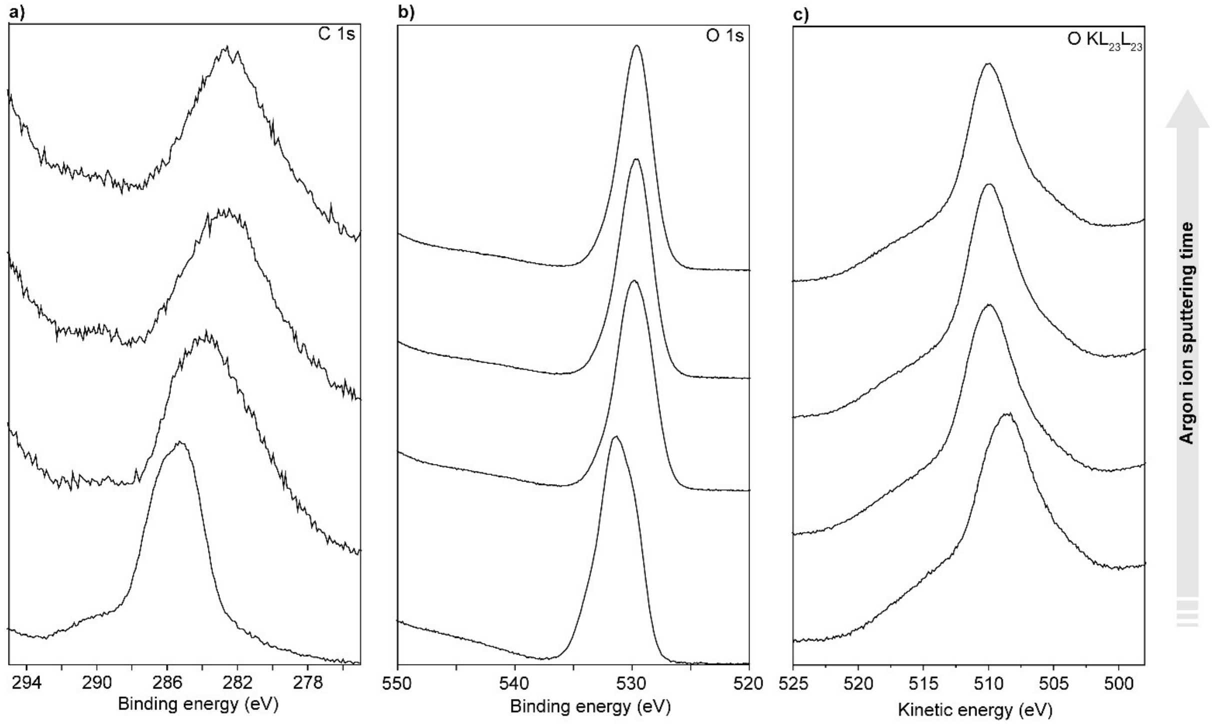

Figure S9. X-ray photoelectron spectroscopy (XPS) measurements for MgO to observe the effect of surface argon sputtering over time for **a-c)** C 1s, O 1s, and O KLL. The aim of these measurements was to confirm the assignment of lattice oxygen, hydroxide and carbonate species, as the surface argon sputtering process should completely or partially remove hydroxide and carbonate species, resulting in a decrease of these two species after argon sputtering and an increase in the lattice oxygen signal. The bottom measurement of each figure shows the spectra before argon sputtering, used as a reference. A significant shift to lower binding energy for the C 1s and O 1s spectra, and higher kinetic energy for the O KLL spectra, is observed after the first argon sputtering cycle. This shift suggests a decrease in the content of hydroxide species and an increase in the signal for lattice oxygen.

CeO_2, La_2O_3, Y_2O_3 and MgO supports XPS measurements

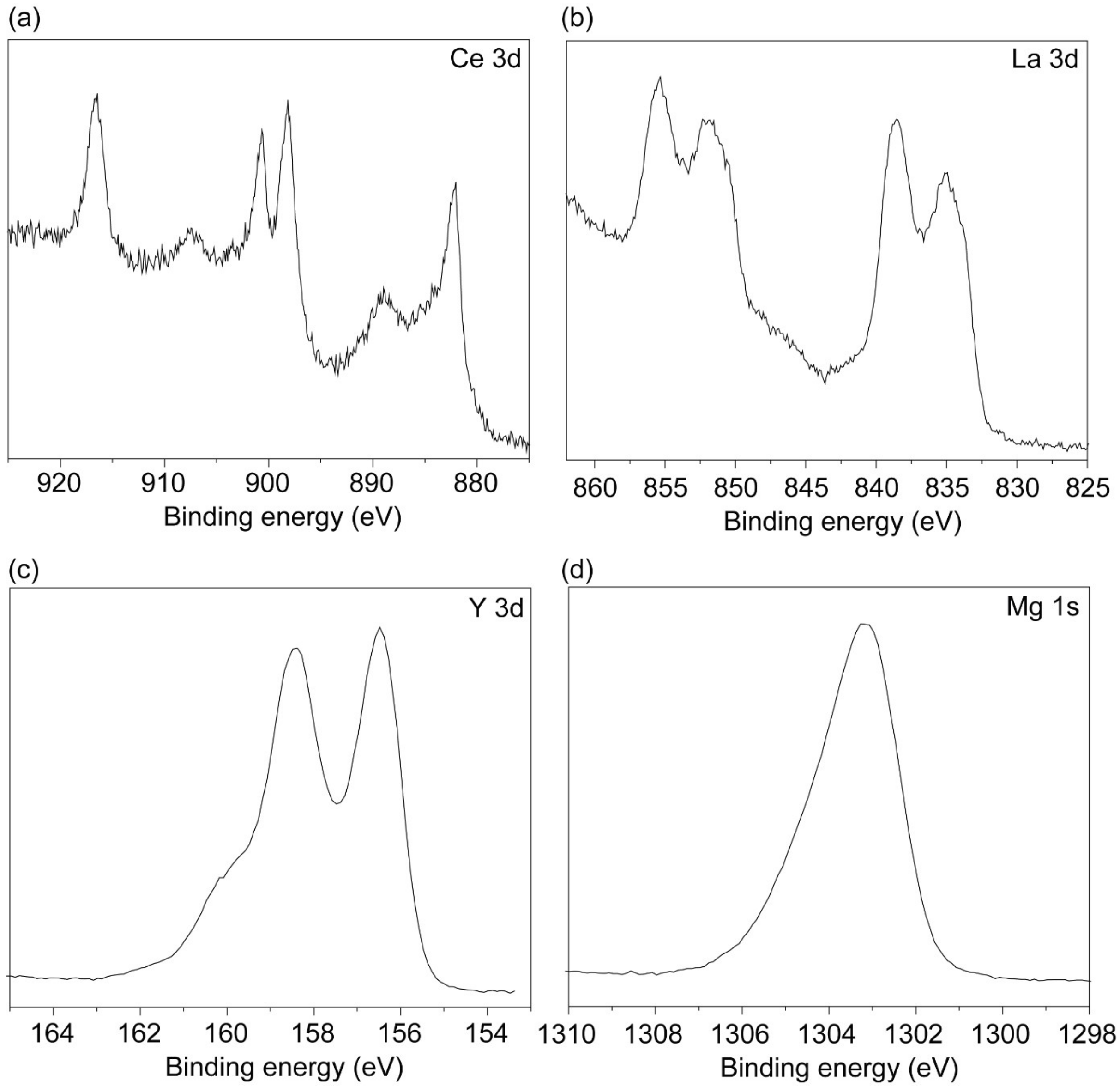

Figure S10. X-ray photoelectron spectroscopy (XPS) measurements of **a-d**) Ce 3d, La 3d, Y 3d and Mg 1s, respectively.

Sc_2O_3 support XPS measurements

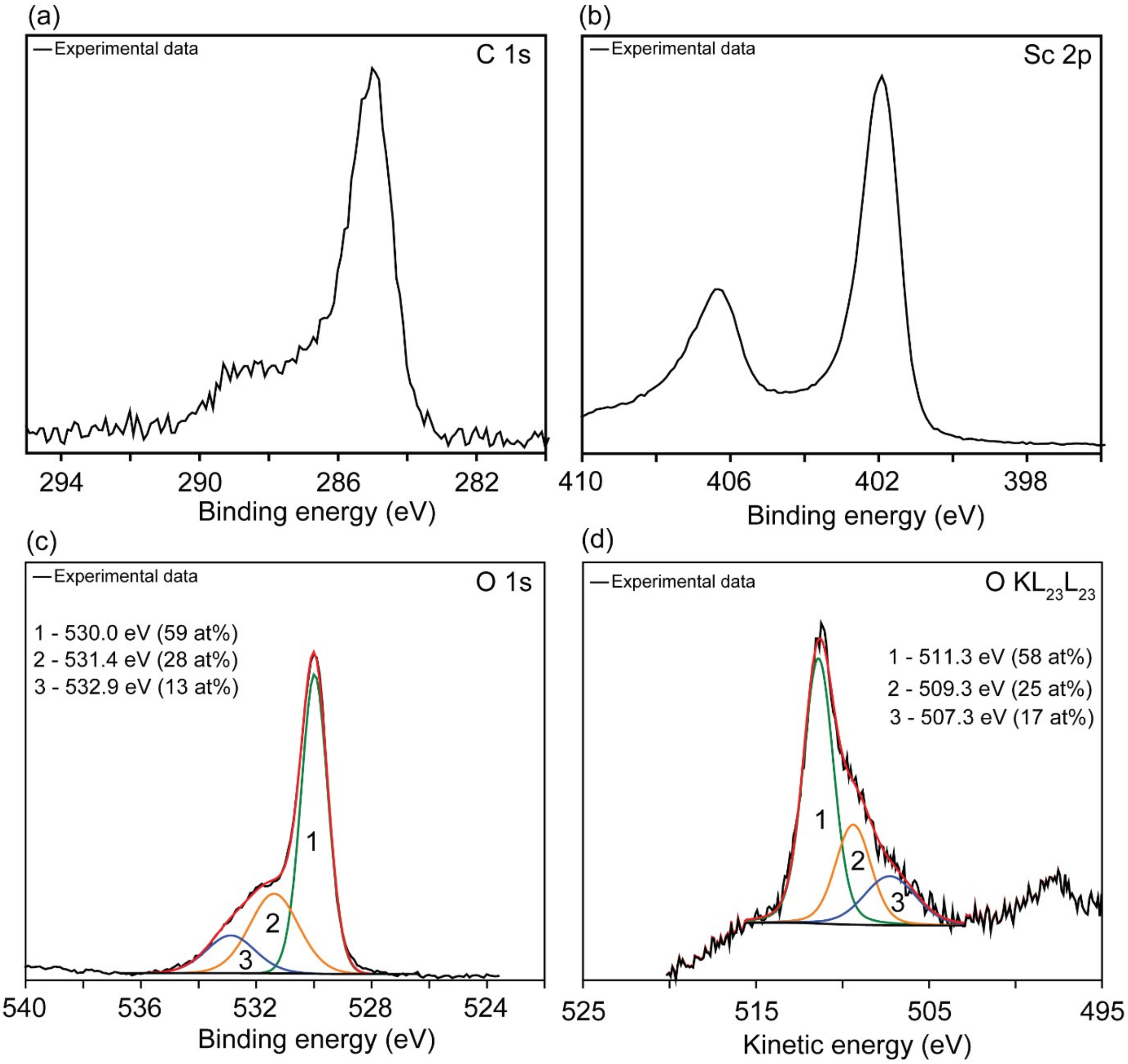

Figure S11. **a-b**) C 1s and Sc 2p spectra for Sc_2O_3. **c-d)** The O 1s and O KLL spectra both show lattice oxygen as the major component, similar to what is observed in CeO_2.

Table S1. Summary of the XPS binding energy and kinetic energy values for the O 1s and O KLL spectra, respectively. The values were averaged from measurements taken at three different locations using Thermo Fisher K-Alpha measurements conducted before argon sputtering.

Supports	O 1s (eV)			$O KL_{23}L_{23}$ (eV)		
	Lattice oxygen	Hydroxide	Carbonate species	Carbonate species	Hydroxide	Lattice oxygen
CeO_2	529.4 ± 0.1	531.2 ± 0.1	532.4 ± 0.3	509.5 ± 0.3	511.0 ± 0.2	513.4 ± 0.1
La_2O_3	529.0 ± 0.1		531.5 ± 0.3	509.8 ± 0.3	-	513.4 ± 0.1
Y_2O_3	529.4 ± 0.1		531.8 ± 0.2	509.2 ± 0.1	-	512.2 ± 0.2
Sc_2O_3	530.0 ± 0.1	531.4 ± 0.1	532.9 ± 0.2	507.3 ± 0.2	509.3 ± 0.2	511.3 ± 0.1
MgO	529.6 ± 0.1	531.6 ± 0.1	-	-	508.1 ± 0.1	510.2 ± 0.1

3. Thermal programmed desorption (TPD) of CO_2

$$d = \frac{6000}{\rho_{He} \cdot A_{BET}}$$

Table S2. Metal oxides A_{BET} surface area for CO_2 desorption measurements.

Entry	Support	Sample mass (mg)	BET surface area ($m^2 g^{-1}$)	Basic sites no reduction (μmol m^{-2})	Basic sites reduction (μmol m^{-2})
1	Y_2O_3	23	15.7	3.3	3.3
2	La_2O_3	30	13.7	2.0	1.8
3	CeO_2	15	77.7	0.3	1.7
4	Sc_2O_3	48	6.7	1.1	1.4
5	MgO	40	116.3	0.4	0.7

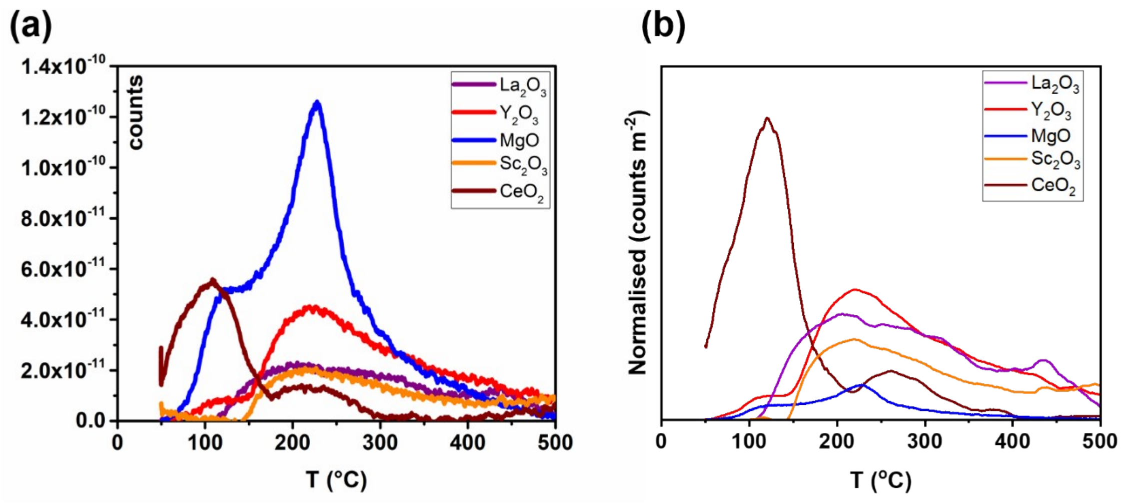

Figure S12. CO_2 desorption from metal oxide supports after baseline correction: (**a**) data without normalisation to the support surface area and (**b**) data normalised to the support surface area, used to calculate CSD values.

The concentrations of Lewis base site (LBS) were measured under two conditions: with and without H_2 reduction prior CO_2 desorption experiments for all samples (see results in Table S2). CeO_2 was the only metal oxide that exhibited significant changes, while no notable changes were observed for the other metal oxides (Table S2). This behaviour is attributed to CeO_2's high reducibility under hydrogen and/or vacuum environments.[4] Since the Ru atoms were deposited under vacuum conditions (with a background pressure of 3×10^{-8} torr and working pressure under argon of 3×10^{-3} torr), the metal atoms landed on a highly reduced metal oxide surface. Therefore, we used the CO_2 TPD results with prior H_2 reduction to mimic the vacuum conditions of the Ru nanoclusters formation. This approach showed very good agreement with our catalysis findings.

4. Ammonia synthesis

Table S3. Catalyst and support masses for catalytic testing. All catalytic materials were furthermore diluted in 2.3 g of SiC.

Entry	Catalyst	Support density (g/L)	Mass support with Ru / mg	Volume Ru on support / mL	Added support mass / mg	Added support V / mL	Ru /mg
1	Ru/CeO_2	65	115	1.700	0	0	1.7
2	Ru/Y_2O_3	543	223	0.412	591	1.09	1.7
3	Ru/MgO	170	100	0.590	195	1.03	1.7
4	Ru/La_20_3	77	85	1.104	39	0.51	1.7
5	Ru/Sc_2O_3	650	196	0.300	910	1.40	1.7

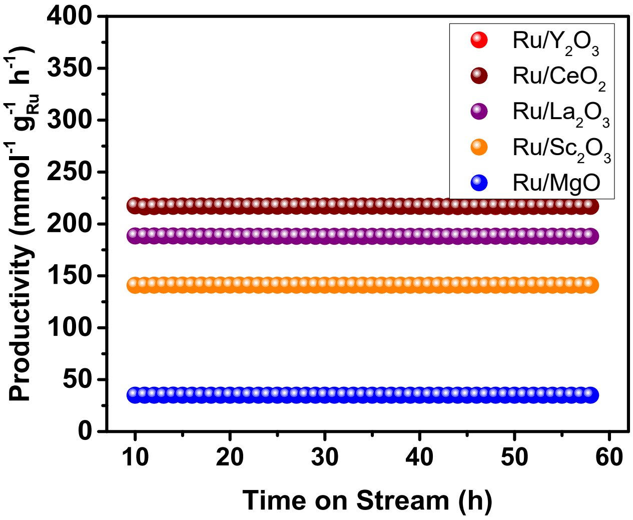

Figure S13. Productivities of Ru onto Sc_2O_3, MgO, La_2O_3, CeO_2 and Y_2O_3 for 58 h on stream.

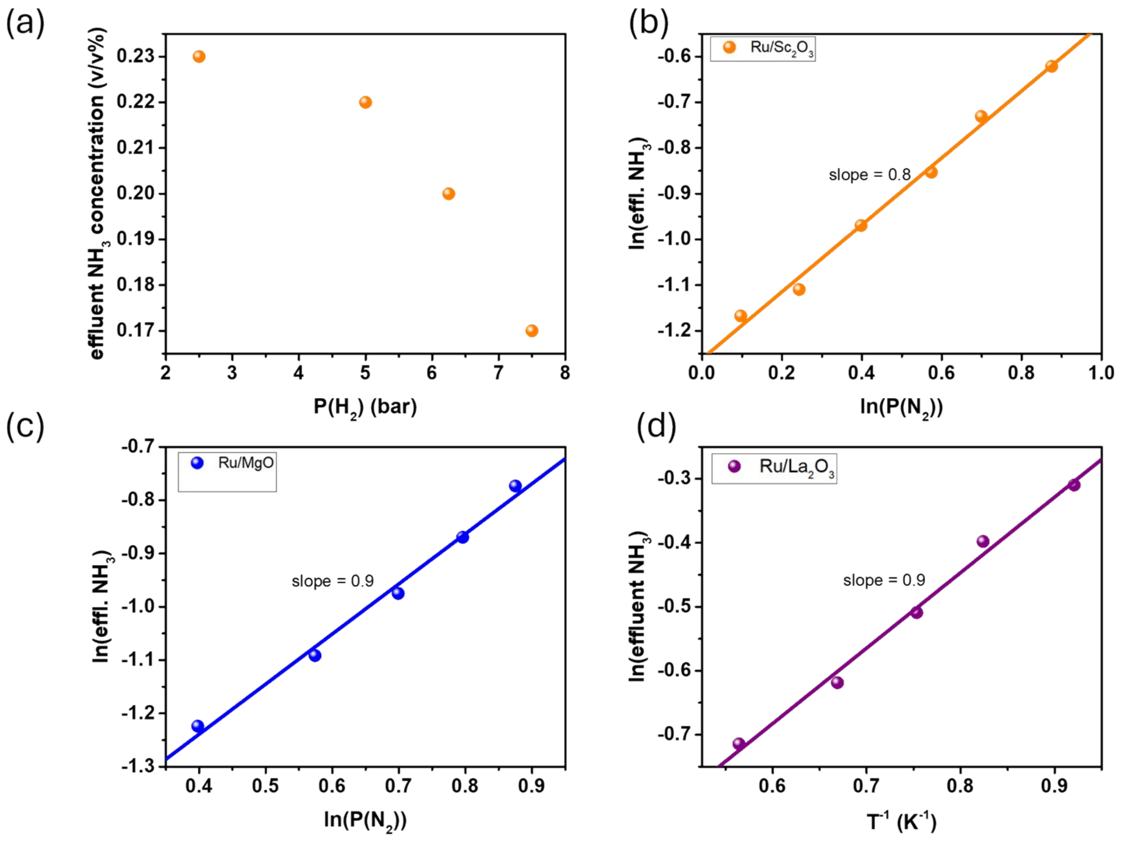

Figure S14. (**a**) Effect of hydrogen on Ru@Sc_2O_3 not presented catalysts in the main paper, **b**-**d**) N_2 order for the not presented catalysts in the main paper.

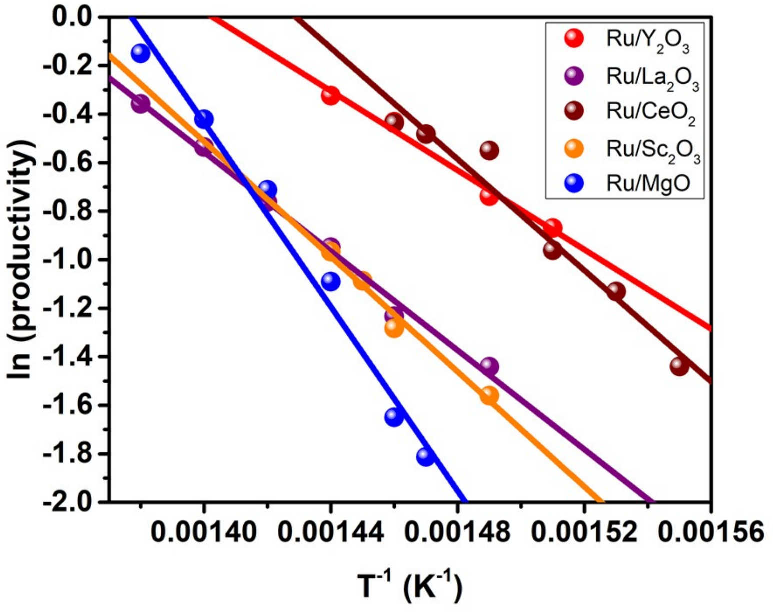

Figure S15. Arrhenius Plot for Ru/CeO_2, Ru/Y_2O_3, Ru/Sc_2O_3, Ru/La_2O_3 and Ru/MgO.

N* binding strength

For TPD analysis, methods developed elsewhere were used.[5] In short, the coverage of N* was assumed to be 1 and the system is considered to reach equilibrium. In accordance with previous reports, the pre-exponential factor *A* was assumed to be $1.5 \cdot 10^{15}$ s^{-1}.[6]

For TPA analysis, the rate constant for adsorption (k_{ads}) the following rate law was assumed:

$$r = k_{ads} P_{N2} \theta_*^2$$

Under initial conditions, where the desorption of N_2 from the surface can be neglected, θ_* can be calculated as:

$$\theta_* = \theta_{tot} - \theta_{N*}$$

Hereby θ_{N*} can be calculated from the amount of N_2 adsorbed onto the Ru surface.

The equilibrium constant for nitrogen adsorption can thus be calculated for varying temperatures because by definition:

$$K_{eq}(T) = \frac{k_{ads}(T)}{k_{des}(T)}$$

Using values of K_{eq}, *ΔH* and *ΔS* can be derived from the Van't Hoff equation.

Additional discussion:

Traditionally N* binding strength is used as a descriptor to predict the catalytic activity of a surface in ammonia synthesis.[7-9] Experimentally the N* binding strength can be determined by temperature programmed adsorption (TPA), temperature programmed desorption (TPD) and $^{15}N_2$:$^{14}N_2$ scrambling experiments.[6, 10-14] Hereby TPA experiments measure the rate constant for dissociative nitrogen adsorption (k_{ads}),[6, 14] whilst from TPD experiments the rate constant for the desorption process can be extracted (k_{des}).[5, 6, 10, 15, 16] As by definition the equilibrium constant (K_{eq}) is defined by the fraction of the adsorption divided by the desorption rate constant, equilibrium constant can be solved and ΔG, ΔH and ΔS can be obtained for the dissociative nitrogen adsorption.

As Y_2O_3, CeO_2 and MgO span the entire width of the catalyst support descriptor (CSD) values determined previously, Ru/Y_2O_3, Ru/CeO_2 and Ru/MgO were chosen to determine the nitrogen bonding strengths. In general, the activation energy of adsorption decreases from Ru/MgO to Ru/CeO_2 and Ru/Y_2O_3 whilst the activation energy for the desorption is within the same range for all samples (Ru/Y_2O_3 has a slightly smaller activation energy of desorption). Interestingly, the decrease in the activation energy of dissociative adsorption and the CSD displays a linear correlation. It is important to note that for Ru/CeO_2 and Ru/MgO N_2 TPD experiments were – in contrast to Ru@Y_2O_3 - not successful but instead TPD of spent catalyst after performing ammonia synthesis were analysed. The difference in acquisition method might explain the difference in between the desorption activation energies observed Ru/Y_2O_3 and Ru/CeO_2, respectively Ru/MgO. Furthermore, the spent catalysts (Ru/MgO and Ru/CeO_2) displayed a secondary desorption process at higher temperatures, which was disregarded for this analysis. To analyse the acquired TPD data, we used a published program and thus we had to made an assumption that the coverage of N* is assumed to be 1 which in reality is not the case.[5]

The obtained values are in general in good agreement with previous reports. [6, 13, 17] For instance, the activation energy of desorption is in the same range as previously reported for Ru/MgO and Ru/C12A7:O^{2-} but as expected significantly higher as reported to electride materials.[6, 13, 17] The pre-exponential factor found for the dissociative adsorption of nitrogen is a magnitude higher as reported previously for Ru/MgO, but still represent very low values indicating a highly activated process for the activation of nitrogen.[6] Finally, the activation energies found for the Ru/MgO prepared by wet chemical deposition but fall again in the same range as reported for Ru@C12A7:O^{2-}.[6, 13, 17]

Using the Arrhenius parameters developed in the previous section, k_{ads} and k_{des} can be calculated at various temperatures and consequently ΔH and ΔS (and thus ΔG^0) can be estimated for the dissociative adsorption of N_2 on the investigated catalysts. As expected, by the lowering activation energy found for the adsorption (whilst desorption activation energies remained constant), the ΔH decreases from Ru/MgO (-12.3 kJ mol^{-1}) over Ru/CeO_2 (-62.7 kJ mol^{-1}) to Ru/Y_2O_3 (-82.8 kJ mol^{-1}), indicating a stronger heat of adsorption in the order. This finding is also reflected in the calculated ΔG^0 values, reflective of a stronger binding strength. Compared to reported values, these values are lower as reported for Ru/MgO prepared by wet chemical methods.[6] For instance, ΔH has been reported to be -97.4 kJ mol^{-1}, whilst the values found here range from -12.3 kJ mol^{-1} to -82.8 kJ mol^{-1}, thus ΔG^0 values determined for our catalysts are in 10 kJ mol^{-1} to 15 kJ mol^{-1} higher.[6] The difference between the determined values in this work are most likely to be associated with the methodology used to determine the Arrhenius parameters of the desorption process (as mentioned earlier a coverage of 1 is assumed).[5, 6] It is important to note that the ΔH values found here are in the same range found previously for Ru/C12A7:O^{2-}.[17]

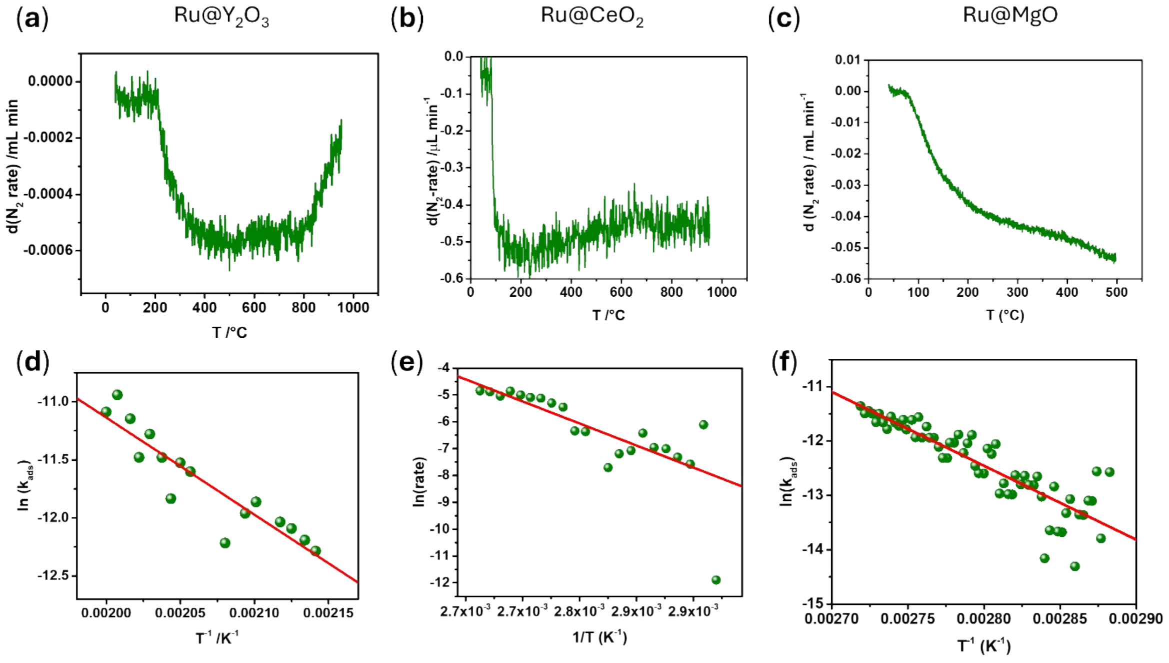

Figure S16. Temperature programmed adsorption of N_2 on for (**a**) Ru/Y_2O_3, (**b**) Ru/CeO_2 and (**c**) Ru/MgO. Arrhenius plots for (**a**) Ru/Y_2O_3, (**e**) Ru/CeO_2 and (**c**) Ru/MgO.

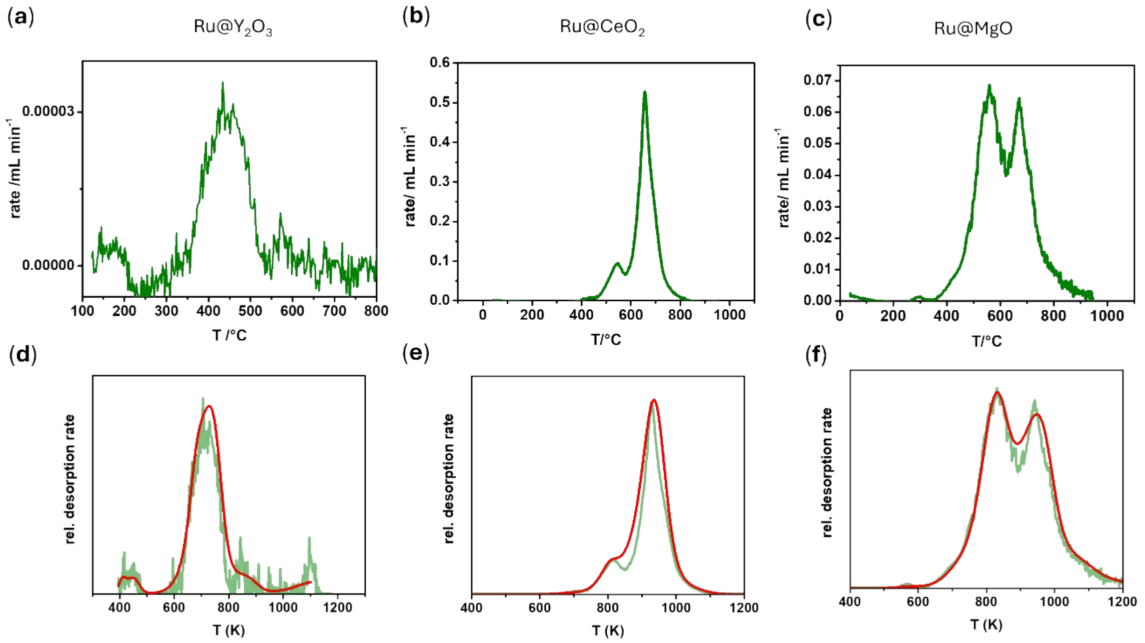

Figure S17. Temperature programmed desorption of N_2 on for (**a**) Ru/Y_2O_3, (**b**) Ru/CeO_2 and (**c**) Ru@MgO. Comparison with simulated desorption TPD spectra (red) for (**a**) Ru/Y_2O_3, (**e**) Ru/CeO_2 and (**c**) Ru/MgO. Spectra are simulated with according to methods described elsewhere.[5]

Table S4. Kinetic results from temperature programmed adsorption (TPA) and temperature programmed desorption (TPD) results.

		Forward reaction		Reverse reaction		
Entry	Catalyst	A ($kPa^{-1}s^{-1}$)	Ea ($kJ\ mol^{-1}$)	A (s^{-1})	Ea ($kJ\ mol^{-1}$)	comment
1	Ru/Y_2O_3	410	97.6 ± 10	$1.5 \cdot 10^{10}$	151.4	This work
2	Ru/CeO_2	263	69 ± 12	$1.5 \cdot 10^{10}$	160.2	This work
3	Ru/MgO	112	152.9 ± 18	$1.5 \cdot 10^{10}$	165.3	This work
4	Ru/MgO	56	60.9	$1.5 \cdot 10^{10}$	158	Previous work[6]
5	Cs-Ru/MgO	56	33	$2.0 \cdot 10^{10}$	137	Previous work[6]
6	Ru/C12A7:e^-	n.a.	n.a.	n.a.	64	Previous work[13]
7	Ru/C12A7:O^{2-}	n.a.	104	n.a.	133	Previous work[17]

Table S5. Thermodynamic parameters found for N_2 adsorption according to data represented in **Table S4**.

Entry	Catalyst	ΔH (kJ mol^{-1})	ΔS (J K^{-1} mol^{-1})	ΔG^0 (kJ mol^{-1})	comment
1	Ru/Y_2O_3	-82.8	-148.5	-39.0	This work
2	Ru/CeO_2	-62.7	-144.8	-19.5	This work
3	Ru/MgO	-12.3	-161.3	36	This work
4	Ru/MgO^b	-97.4	-161	-49.4	Previous work[6]
5	Ru/C12A7:O^{2-}	-29	n.a.	n.a.	Previous work[17]

5. Rate controlling typical for the Sabatier optimum

In general, the mechanistic transformation of N_2 to NH_3 can be divided into two steps. The first step consists of the dissociative adsorption of N_2, whilst the second step is the formation of NH_3^* (via step wise protonation) and the consecutive desorption of NH_3.[18, 19] As the activation barrier for both steps can be related to the binding strength of N* (atomic nitrogen bound to the catalysts surface), it can be deduced that a low binding strength of N* (high catalyst support descriptor (CSD) values) results in a low coverage of N*, thus limiting the rate of transformation to the dissociative adsorption of N_2.[9, 19-22] In this scenario, increasing the partial pressure of H_2 leads to an increased coverage with H*, removing available sites for N* thus reducing the rate of transformation. Consequently, an order ≤0 can be expected for H_2.[7] On the other extreme, N* displays a high binding strength (low CSD values), thus a high coverage of N* is to be expected and thus the rate of transformation is limited to the consumption of N* (the step wise protonation of N* to NH_3^* and release of NH_3^*. Here an order ≥0 for H_2 is to be expected as the coverage of H* is increased thus facilitating the protonation of N*.[7, 18, 19]
Near or at the Sabatier optimum a clear determination of the rate determined step (RDS) is not possible but should rather be investigated by the degree of rate control.[18, 19, 23-26] Whilst with catalysts far from the Sabatier optimum the RDS can usually be subscribed to a single step (the degree rate control is $X_{DRC} \approx 1$ for one step), near or at the Sabatier optimum the rate of transformation is defined by two or more steps and the degree of rate control is not near unity for either of the limiting steps.[18, 24-26] Furthermore, the degree of rate control is dependent on the applied reaction conditions, such as a pressure and temperature etc.[19, 23, 26] Applied to the ammonia synthesis, this translates to a relatively low concentration (high compared to catalysts with weak binding strengths of N*) at low partial pressures of N_2 and high partial pressure of H_2.[7] Here the overall transformation is limited by the dissociative adsorption of N_2 and the order of H_2 is ≤0 but when the partial pressure of H_2 is decreased, the coverage of N* increases and the stepwise protonation N* becomes rate controlling, thus resulting in a rate order ≥0 for H_2.[7, 9, 18, 24, 26]
Both the study of the apparent activation Energy (E_{app}) in dependence of the CSD developed in this work and the study of the partial pressure onto the overall rate of transformation suggest that our descriptor can be used analogously to the binding strength of N* used in earlier studies.[7, 9, 19-22] For

instance, E_{app} of 76 kJ mol^{-1} and the fluctuation in the degree in rate control observed for Ru@Y_2O_3, suggest that Ru@Y_2O_3 possess electronic properties close to those expected for a catalyst near to the Sabatier optimum.[7, 18, 24, 26, 27] Finally, increasing the CSD values lead to a linearly increased of E_{app} values and the dissociative N_2 adsorption becomes rate controlling (as indicated by the effect of partial pressure of H_2 onto the rate) with a similar behaviour to be expected for lowering the binding strength of N*.[7]

6. Estimation of Surace sites in Ru nanoclusters

All calculations assume spheres for the here investigated Ru nanoclucsters.

The volume of a Ru nanocluster is given by

$$V = \frac{\pi d^3}{6}$$

Where d is the diameter of a given nanocluster. The volume of the sub-surface layer of a nanocluster can be calculated accordingly as:

$$V = \frac{\pi (d - 2d_{Ru})^3}{6}$$

Where d_{Ru} is the atomic diameter of a Ru atom. The density of Ru is given by 12.364 g cm^{-3}. Therefore, there are 73.67 atoms nm^{-3}. Consequently, the number of atoms in a whole nanocluster and the subsurface can be calculated. With both values the surface Ru atoms can be calculated according to:

$$Ru_{surf} = Ru_{NP} - Ru_{sub}$$

Where Ru_{surf} are the Ru atoms on the NP surface; Ru_{Np} are the Ru atoms in the whole nanocluster and Ru_{sub} are the Ru atoms sub surface. To validate our findings using the diameter of the Ru NP we conducted CO pulse sorption experiments with Ru/CeO_2 and Ru/MgO. Both validation experiments confirm the dispersion found via the nanocluster diameter. The table below summarises all the results.

For a hemisphere, both the total volume and the volume of the subsurface are halved. Note the difference between assuming a sphere and a hemisphere: assuming a sphere does not significantly change the dispersion levels.

Table S6. Dispersion of the Ru nanocluster on the here investigated catalytic materials.

Entry	Catalyst	Ru NP diameter (nm)	Dispersion by diameter (sphere)	Dispersion by diameter (hemisphere)	Dispersion by CO pulse sorption
1	Ru/MgO	2.2	0.57	0.59	0.61
2	Ru/CeO_2	2.3	0.55	0.55	0.56
3	Ru/Y_2O_3	2.3	0.55	0.55	-
4	Ru/La_2O_3	3.4	0.40	0.40	-
5	Ru/Sc_2O_3	1.8	0.65	0.65	-

7. Pre-exponential factors and other effects affecting the overall rate

Table S7. Pre-exponential factors found for the catalysts.

Entry	Catalyst	ln(A)
1	Ru/Y_2O_3	11.5
2	Ru/La_2O_3	13.7
3	Ru/CeO_2	16.4
4	Ru/Sc_2O_3	16.1
5	Ru/MgO	26.1

It is worth pointing out that the concentration of surface sites within the samples varies, which manifests itself in a differing pre-exponential factor. Furthermore, the pre-exponential factor depends on the concentration of catalytic intermediates on the Ru surface, which also varies. These two factors explain the difference in pre-exponential, as found in Table S7.

8. Comparison of Ru nanoclusters before and after the reaction

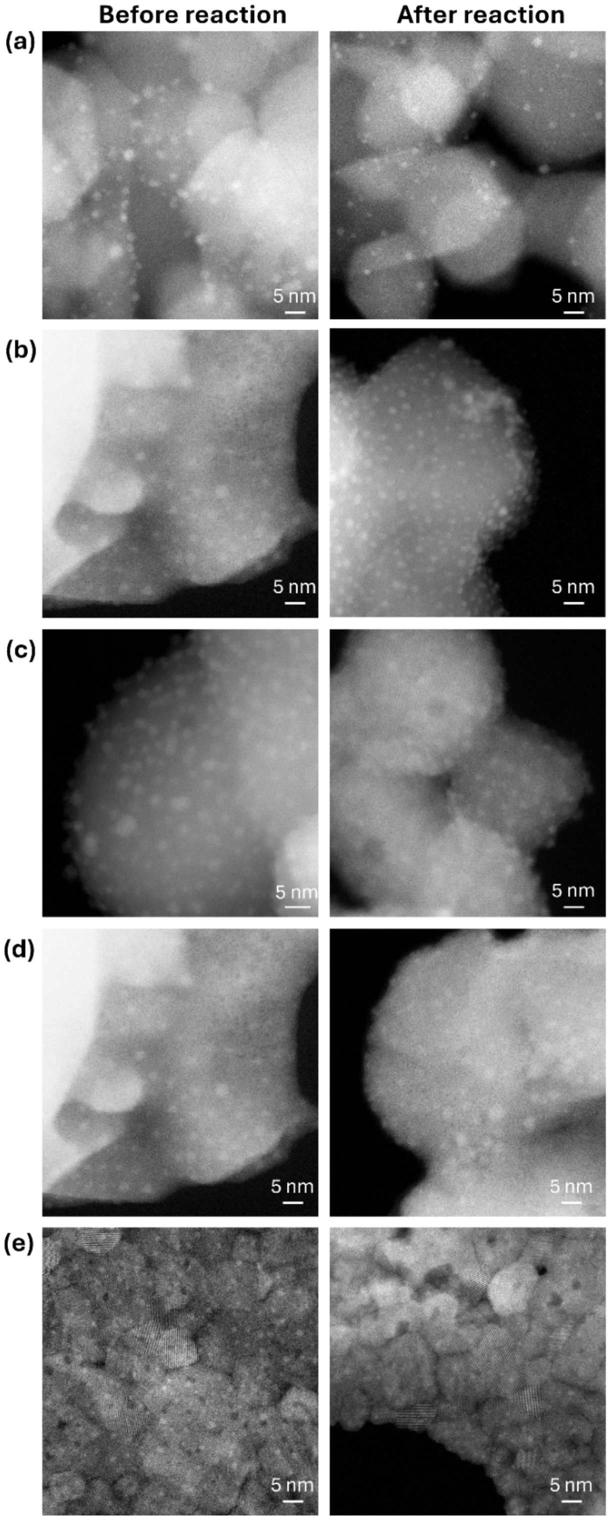

Figure S18. Comparison of Ru nanoclusters deposited onto (**a**) MgO, (**b**) Sc_2O_3 (**c**) Y_2O_3, (**d**) La_2O_3 and (**e**) CeO_2 before and after ammonia synthesis at 400°C and 20 bar for 58h. No significant changes in the Ru particle size are observed.

8. References

1. Popov, I.; Plekhanov, E.; Tchougréeff, A.; Besley, E., Effective hamiltonian of crystal field method for periodic systems containing transition metals. *Molecular Physics* **2023,** *121* (9-10), e2106905.
2. Morgan, D. J., Photoelectron spectroscopy of ceria: Reduction, quantification and the myth of the vacancy peak in XPS analysis. *Surface and Interface Analysis* **2023,** *55* (11), 845-850.
3. Sunding, M. F.; Hadidi, K.; Diplas, S.; Løvvik, O. M.; Norby, T. E.; Gunnæs, A. E., XPS characterisation of in situ treated lanthanum oxide and hydroxide using tailored charge referencing and peak fitting procedures. *Journal of Electron Spectroscopy and Related Phenomena* **2011,** *184* (7), 399-409.
4. Lin, Y.; Wu, Z.; Wen, J.; Poeppelmeier, K. R.; Marks, L. D., Imaging the Atomic Surface Structures of CeO2 Nanoparticles. *Nano Letters* **2014,** *14* (1), 191-196.
5. Schmid, M.; Parkinson, G. S.; Diebold, U., Analysis of Temperature-Programmed Desorption via Equilibrium Thermodynamics. *ACS Physical Chemistry Au* **2023,** *3* (1), 44-62.
6. Hinrichsen, O.; Rosowski, F.; Hornung, A.; Muhler, M.; Ertl, G., The Kinetics of Ammonia Synthesis over Ru-Based Catalysts: 1. The Dissociative Chemisorption and Associative Desorption of N2. *Journal of Catalysis* **1997,** *165* (1), 33-44.
7. Dahl, S.; Logadottir, A.; Jacobsen, C. J. H.; Nørskov, J. K., Electronic factors in catalysis: the volcano curve and the effect of promotion in catalytic ammonia synthesis. *Applied Catalysis A: General* **2001,** *222* (1), 19-29.
8. Boisen, A.; Dahl, S.; Nørskov, J. K.; Christensen, C. H., Why the optimal ammonia synthesis catalyst is not the optimal ammonia decomposition catalyst. *Journal of Catalysis* **2005,** *230* (2), 309-312.
9. Bligaard, T.; Nørskov, J. K.; Dahl, S.; Matthiesen, J.; Christensen, C. H.; Sehested, J., The Brønsted–Evans–Polanyi relation and the volcano curve in heterogeneous catalysis. *Journal of Catalysis* **2004,** *224* (1), 206-217.
10. Rosowski, F.; Hinrichsen, O.; Muhler, M.; Ertl, G., The temperature-programmed desorption of N2 from a Ru/MgO catalyst used for ammonia synthesis. *Catalysis Letters* **1996,** *36* (3), 229-235.
11. Fastrup, B., Temperature-Programmed Adsorption and Desorption of Nitrogen on Iron Ammonia Synthesis Catalysts. *Journal of Catalysis* **1994,** *150* (2), 345-355.
12. Ertl, G., Surface Science and Catalysis—Studies on the Mechanism of Ammonia Synthesis: The P. H. Emmett Award Address. *Catalysis Reviews* **1980,** *21* (2), 201-223.
13. Hayashi, F.; Toda, Y.; Kanie, Y.; Kitano, M.; Inoue, Y.; Yokoyama, T.; Hara, M.; Hosono, H., Ammonia decomposition by ruthenium nanoparticles loaded on inorganic electride C12A7:e−. *Chemical Science* **2013,** *4* (8), 3124-3130.
14. Kuganathan, N.; Hosono, H.; Shluger, A. L.; Sushko, P. V., Enhanced N2 Dissociation on Ru-Loaded Inorganic Electride. *Journal of the American Chemical Society* **2014,** *136* (6), 2216-2219.
15. Wilmer, H.; Genger, T.; Hinrichsen, O., The interaction of hydrogen with alumina-supported copper catalysts: a temperature-programmed adsorption/temperature-programmed desorption/isotopic exchange reaction study. *Journal of Catalysis* **2003,** *215* (2), 188-198.
16. Xu, J.; Deng, J., Theoretical Parameter-Free Analysis Model for Temperature-Programmed Desorption (TPD) Spectra. *ACS Omega* **2020,** *5* (8), 4148-4157.
17. Hara, M.; Kitano, M.; Hosono, H., Ru-Loaded C12A7:e– Electride as a Catalyst for Ammonia Synthesis. *ACS Catalysis* **2017,** *7* (4), 2313-2324.
18. Motagamwala, A. H.; Dumesic, J. A., Chemical kinetics for generalized two-step reaction schemes. *Journal of Catalysis* **2021,** *404*, 850-863.
19. Kozuch, S.; Shaik, S., Kinetic-Quantum Chemical Model for Catalytic Cycles: The Haber–Bosch Process and the Effect of Reagent Concentration. *The Journal of Physical Chemistry A* **2008,** *112* (26), 6032-6041.

20. Abild-Pedersen, F.; Greeley, J.; Studt, F.; Rossmeisl, J.; Munter, T. R.; Moses, P. G.; Skúlason, E.; Bligaard, T.; Nørskov, J. K., Scaling Properties of Adsorption Energies for Hydrogen-Containing Molecules on Transition-Metal Surfaces. *Physical Review Letters* **2007,** *99* (1), 016105.
21. Vojvodic, A.; Medford, A. J.; Studt, F.; Abild-Pedersen, F.; Khan, T. S.; Bligaard, T.; Nørskov, J. K., Exploring the limits: A low-pressure, low-temperature Haber–Bosch process. *Chemical Physics Letters* **2014,** *598*, 108-112.
22. Medford, A. J.; Vojvodic, A.; Hummelshøj, J. S.; Voss, J.; Abild-Pedersen, F.; Studt, F.; Bligaard, T.; Nilsson, A.; Nørskov, J. K., From the Sabatier principle to a predictive theory of transition-metal heterogeneous catalysis. *Journal of Catalysis* **2015,** *328*, 36-42.
23. Kozuch, S.; Shaik, S., A Combined Kinetic−Quantum Mechanical Model for Assessment of Catalytic Cycles: Application to Cross-Coupling and Heck Reactions. *Journal of the American Chemical Society* **2006,** *128* (10), 3355-3365.
24. Campbell, C. T., The Degree of Rate Control: A Powerful Tool for Catalysis Research. *ACS Catalysis* **2017,** *7* (4), 2770-2779.
25. Mao, Z.; Campbell, C. T., Apparent Activation Energies in Complex Reaction Mechanisms: A Simple Relationship via Degrees of Rate Control. *ACS Catalysis* **2019,** *9* (10), 9465-9473.
26. Campbell, C. T.; Mao, Z., Analysis and prediction of reaction kinetics using the degree of rate control. *Journal of Catalysis* **2021,** *404*, 647-660.
27. Feng, J.; Liu, L.; Zhang, X.; Wang, J.; Ju, X.; Li, R.; Guo, J.; He, T.; Chen, P., Ru nanoparticles on Y2O3 with enhanced metal–support interactions for efficient ammonia synthesis. *Catalysis Science & Technology* **2023,** *13* (3), 844-853.
